# Antiradical Activity of Dopamine, L-DOPA, Adrenaline, and Noradrenaline in Water/Methanol and in Liposomal Systems

**DOI:** 10.1021/acs.joc.1c02308

**Published:** 2021-12-06

**Authors:** Katarzyna Jodko-Piórecka, Bożena Sikora, Monika Kluzek, Paweł Przybylski, Grzegorz Litwinienko

**Affiliations:** † Faculty of Chemistry, University of Warsaw, Pasteura 1, 02-093 Warsaw, Poland; ‡ Laboratory of Biological Physics, Institute of Physics, Polish Academy of Sciences, Al. Lotnikow 32/46, 02-668 Warsaw, Poland; § Department of Materials and Interfaces, Weizmann Institute of Science, Rehovot 76100, Israel

## Abstract

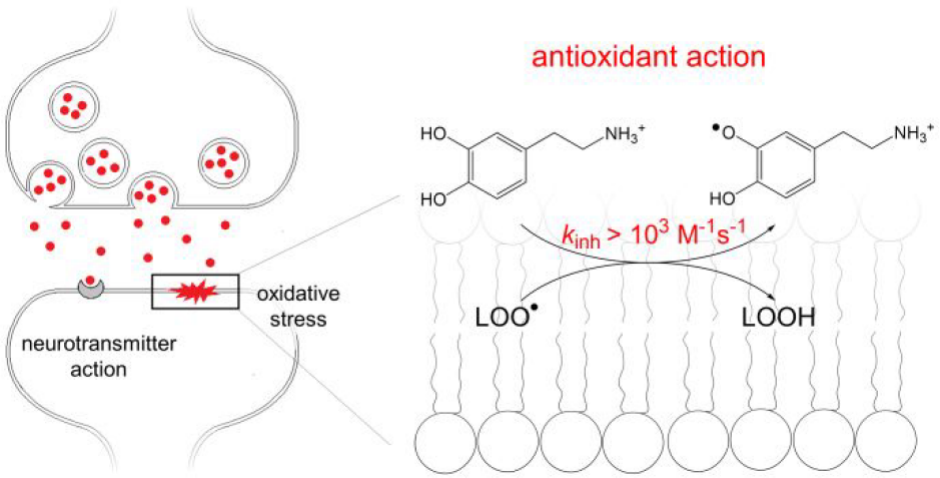

Catecholamines play a crucial role in signal transduction and are also expected to act as endogeneous antioxidants, but the mechanism of their antioxidant action is not fully understood. Here, we describe the impact of pH on the kinetics of reaction of four catecholamines (L-DOPA, dopamine, adrenaline, and noradrenaline) with model 2,2-diphenyl-1-picrylhydrazyl radical (dpph^•^) in methanol/water. The increase in pH from 5.5 to 7.4 is followed by a 2 order of magnitude increase in the rate constant, e.g., for dopamine (DA) *k*^pH5.5^ = 1,200 M^–1^ s^–1^ versus *k*^pH7.4^ = 170,000 M^–1^ s^–1^, and such rate acceleration is attributed to a fast electron transfer from the DA anion to dpph^•^. We also proved that at pH 7.0 DA breaks the peroxidation chain of methyl linoleate in liposomes assembled from neutral and negatively charged phospholipids. In contrast to no inhibitory effect during peroxidation in non-ionic emulsions, in bilayers one molecule of DA traps approximately four peroxyl radicals, with a rate constant *k*_inh_ >10^3^ M^–1^ s^–1^. Our results from a homogeneous system and bilayers prove that catecholamines act as effective, radical trapping antioxidants with activity depending on the ionization status of the catechol moiety, as well as microenvironment: organization of the lipid system (emulsions vs bilayers) and interactions of catecholamines with the biomembrane.

## Introduction

**Figure 1:**
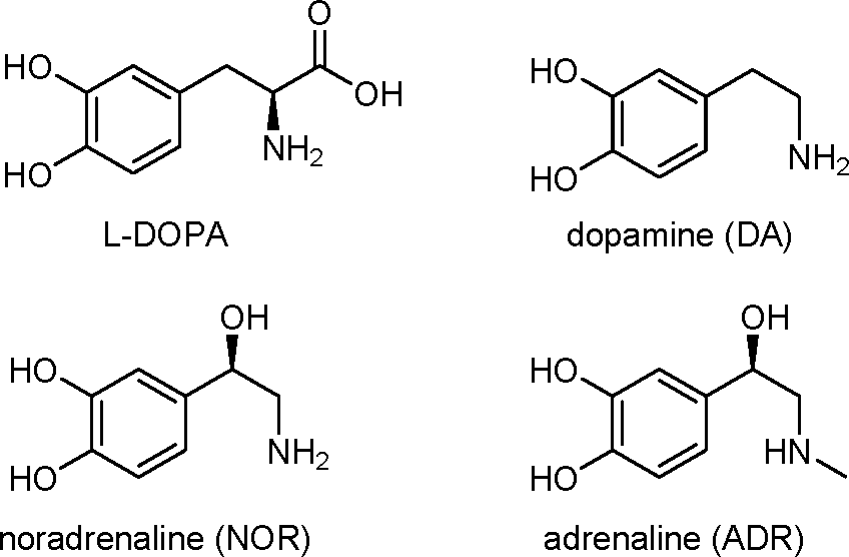
Structures and acronyms of four catecholamines.

Catecholamines (dopamine, adrenaline, noradrenaline, and their precursor L-DOPA, see Figure 1) act as neurotransmitters in the mammalian nervous system^1^ and as hormones in blood circulation. They participate in a variety of motor and mental functions of the organism, and even a slight dysregulation of their activity may lead to pathological events;^1,2^ for example, the motor symptoms in Parkinson’s disease (PD) are associated with severe depletion of dopamine (DA) in the striatum.^3,4^ The results of *in vitro* experiments on neuronal cell lines^5^ and peripheral blood cells^6^ suggest that catecholamines might also act as endogenous antioxidants, because their protective effect against cell death^5^ is associated with a decrease in the concentration of the intracellular reactive oxygen species (ROS)^5a^ and can be mimicked by other antioxidants, e.g., analogues of α-tocopherol,^5a,5b^ catechol derivatives like 3,4-dihydroxymandelic acid (the product of noradrenaline metabolism), and catechol itself.^5a^ In contrast, l-tyrosine (monohydroxyphenol) and normetanephrine (O-methylated noradrenaline) do not protect neuronal lines against cell death,^5a^ suggesting the crucial role of the catechol moiety in neuroprotective activity of catecholamines.^5a^ This is not surprising because catechols are responsible for the excellent antioxidant activity of many natural compounds,^7^ including flavonoids^7f,7g,8^ and phenylpropanoids.^8^

In contrast to many papers describing the impact of catechols and catechol derivatives on the level of oxidative stress, and despite the crucial physiological role of catecholamines, their ability to scavenge free radicals has been confirmed only in a few works, including kinetic^9^ and theoretical studies.^10,11^ The first evidence for a direct reaction of catecholamines with an artificial, model free radical [2,2-diphenyl-1-picrylhydrazyl radical (dpph^•^)] was presented nearly three decades ago by Liu and Mori.^12^ More recently, reactions of catecholamines [abbreviated as Ar(OH)_2_] with model cumyloxyl and *tert*-butyloxyl radicals (Y^•^) have been studied by Cosa and Scaiano^9a^ and by Ohkubo et al.^9c^ The latter study successfully employed EPR technique to detect an *o*-semiquinone radical [Ar(OH)O^•^] formed after abstraction of a hydrogen atom from the catechol moiety:
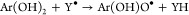
1Cosa and Scaiano^9a^ demonstrated (for Y^•^ = *t-*BuO^•^, in acetonitrile/acetic acid) that <5% of hydrogen abstractions are from the sites other than the catechol moiety.

Reaction 1 may proceed via several mechanisms (including one-step or multistep processes), and the contribution of each mechanism depends on the intrinsic reactivities of both reacting species and the environment [mainly the solvent polarity and its ability to form hydrogen bonds (HBs)]. In acetonitrile and propionitrile, the reaction of catecholamines with alkoxyl (*tert*-butoxyl, galvinoxyl, and cumyloxyl)^9^ and cumylperoxyl^9c^ radicals proceeds via a direct one-step abstraction of a hydrogen atom from catecholamine by the radical, i.e., via a hydrogen atom transfer (HAT) mechanism. HAT is predominant in nonpolar solvents, with formation of the *o*-semiquinone radical that is additionally stabilized by the intramolecular HB formed between oxygen with an odd electron and the hydrogen atom from the adjacent hydroxyl group (OH–O^•^).^13^ Such an intramolecular HB is considerably stronger (∼8 kcal/mol) than the HB in the parent molecule (OH–OH, 4 kcal/mol).^7c^ The presence of metal cations can facilitate a mechanism more complex than HAT, named metal ion-coupled electron transfer (MCET), that was reported for DA (but not for other catecholamines) reacting with galvinoxyl radicals in acetonitrile in the presence of magnesium salts.^9b^

We expect that in polar, ionization-supporting solvents (like water or short chain alcohols) and in biphasic water/lipid systems, more complex mechanisms might occur for all catecholamines, including sequential proton loss electron transfer (SPLET), in which deprotonation of the hydroxyl group is followed by a fast electron transfer from the phenolate anion to an electron deficient radical Y^•^ (like dpph^•^):^13a,14,15^
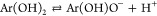
2
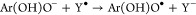
3The role of SPLET in radical scavenging by catecholamines has not been determined so far, despite the preferential location of catecholamines (as hydrophilic molecules, positively charged at physiological pH)^16^ in water or at the water/membrane interface,^17^ i.e., in the microenvironments that support SPLET. Surprisingly, the only publication on the scavenging activity of catecholamines in water and in a lipid environment (but not in the heterogeneous system) is a computational study of DA reactivity toward HO^•^ and HOO^•^ radicals,^10^ indicating HAT and radical adduct formation (RAF) mechanisms in the lipid phase versus a two-step mechanism with separate electron and proton transfer in water. Because this theoretical prediction was not confirmed by any experimental study, we decided to verify the ability of catecholamines to scavenge free radicals in a model lipid/water system, expecting the occurrence of a two-step mechanism, depending on the ionization status of catecholamine. In our previous study, we determined the antioxidant activity of DA and L-DOPA in a simplified heterogeneous system, with lipids dispersed in Triton X-100 micelles.^18^ To our surprise, in this system, catecholamines could not break the chain of lipid peroxidation by scavenging lipid peroxyl radicals, but they showed only retarding activity, arising from their direct reaction with initiating radicals, massively formed in the aqueous phase via initiator decomposition. Although this reaction is less interesting from the physiological perspective than the expected interfacial reaction of catecholamines with peroxyl radicals, it confirms the ability of catecholamines to trap free radicals in water.

Herein, we present the results of our studies on the reaction of four catecholamines [L-DOPA, DA, ADR, and NOR (Figure 1)] with a model dpph^•^ radical in a water/methanol system, in which the protonation status of catecholamine was precisely controlled by the change in pH. Afterward, we verified the antioxidant properties of dopamine (chosen as the most active catecholamine in kinetic studies) in the process of peroxidation of lipid membranes. Experiments were performed on phosphatidylcholine liposomes with the increasing negative surface charge able to attract dopamine toward the membrane surface. The surface charge of one of the studied systems reflected the surface charge of the neuronal lipid membrane. Determination of the antioxidant activity of dopamine in such a model system is an important step toward understanding its potential protective role in the nervous system.

## Results

### Determination of the Acidity Constants p*K*_a_ of Dopamine

Charges of biomolecules play an important role in their activity and localization; thus, we analyzed the possible protonation equilibria before performing the kinetic studies. Catecholamines can easily undergo oxidation to quinones in a pH-dependent manner;^19^ thus, we used point by point analysis, with each scan recorded for a freshly prepared sample, as proposed by Sánchez-Rivera et al.^19a^ to avoid the oxidation of DA during spectrophotometric titration. Analysis of a series of spectra in the pH range of 1.5–13.0 [methanol/water 1:1 (v:v)] gave p*K*_a_ values for DA of 8.37, 10.25, and 12.49, reasonably close to those reported by other authors (see Table S1).

### Kinetics of Reactions of Catecholamines with the dpph^•^ Radical

The method of determination of the rate constants for reaction of catecholamines with dpph^**•**^ was the same as we used previously in our studies of reactivity and solvent effects of some substituted phenols.^14^ The reaction rates were monitored by a stopped-flow technique in water/methanol [1:1 (v:v)] at pH 5.5 and 7.4 at 296 ± 2 K, and kinetic analysis was performed for very initial rates, within tenths of a second after mixing of the solutions of dpph^**•**^ (at a constant initial concentration) with a solution of catecholamine (at minimum six different initial concentrations). To obtain pseudo-first-order conditions, dpph^**•**^ always reacted with a stoichiometric excess of catecholamine. Thus, the experimental pseudo-first-order rate constants, *k*_exp_, plotted against catecholamine concentration gave a straight line *k*_exp_ = *k*^S^[catecholamine] + constant, with the slope *k*^S^ representing the bimolecular rate constant for the reaction of catecholamine with dpph^•^. The values of *k*^S^ determined for DA, L-DOPA, NOR, and ADR are listed in Table 1.

**Table 1:** Bimolecular Rate Constants, *k*^S^, for Reactions of L-DOPA, DA, NOR, and ADR with the dpph^**•**^ Radical in a Water/Methanol System [1:1 (v:v)] at 296 ± 2 K and pH 5.5 and 7.4 and Calculated Degrees of Dissociation (α)

phenol (p*K*_a1_)^a^	pH	α (%)^b^	*k*^S^ (M^–1^ s^–1^)
L-DOPA (8.76)	5.5	0.05	440 ± 30
	7.4	4.20	59000 ± 7000
DA (8.37)	5.5	0.13	1200 ± 200
	7.4	9.69	170000 ± 10000
NOR (8.58)	5.5	0.08	290 ± 30
	7.4	6.24	46000 ± 5000
ADR (8.64)	5.5	0.07	630 ± 60
	7.4	5.46	30000 ± 3000

a p*K*_a_ value for DA determined in this work; for other p*K*_a_ values, see Table S1. For L-DOPA, p*K*_a1_ means deprotonation of the most acidic noncarboxyl group.

b With the assumption that p*K*_a1_ is connected with deprotonation of a first catecholic hydroxyl (see Discussion with described controversies on the protonation order), parameter α describes the fraction of phenolic anions. However, if the first p*K*_a_ is assigned to deprotonation of the alkylammonium cation, the degree of ionization of phenolic hydroxyls will be at least 10 times smaller (see Discussion).

### Antioxidant Activity of Dopamine and PMHC in Model Lipid Systems

Experiments were performed on 100 nm large unilamellar vesicles (LUVs) composed of neutral DMPC or DMPC mixed with anionic DMPG (at molar ratios of 3:1, 1:1, and 1:3) or LUVs composed of pure DMPG. The liposomes were doped with methyl linoleate (MeLin) as an active component undergoing peroxidation. The molar ratio of MeLin to phospholipid was 1:8, which serves as a compromise allowing a sufficient, easy-to-detect accumulation of peroxidation products in time but minimal changes in bilayer organization (as unsaturated lipids have a fluidifying impact on the membrane).^20^ The homogeneous distribution of MeLin in the bilayer was ensured by the method of liposome preparation (see Experimental Section).

**Figure 2:**
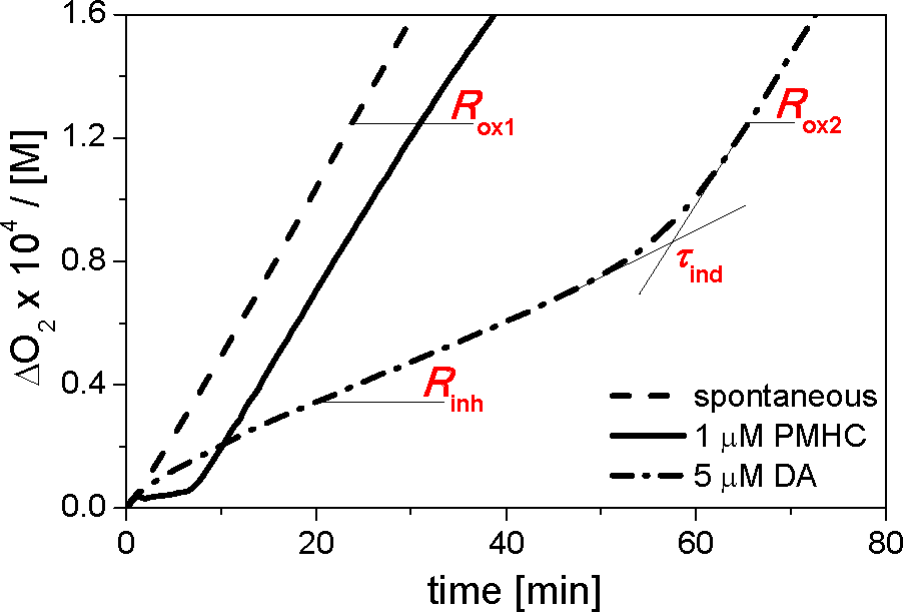
Plots of oxygen uptake, Δ[O_2_], recorded during the peroxidation of 2.74 mM MeLin in LUVs composed of DMPC and DMPG at a molar ratio of 3:1. Uninhibited peroxidation (dashed line, described as “spontaneous” process); peroxidation inhibited by 1 μM PMHC (solid line) or 5 μM DA (dash–dotted line). *R*_ox1_, rate of uninhibited oxidation; *R*_inh_, rate of the process during the inhibition period (the end of this period is indicated as τ_ind_); *R*_ox2_, rate of the postinhibited process (after τ_ind_). The values of parameters *R*_ox1_, *R*_inh_, τ_ind_, and *R*_ox2_ are listed in Table S6. Experiments were performed at 310 K and pH 7.0 with 10 mM ABAP used to initiate the peroxidation of MeLin.

Peroxidation was initiated by a water-soluble azo initiator, 2,2′-azobis(2-methylpropionamidine) dihydrochloride (ABAP), that decomposes in the aqueous phase to positively charged,^21^ carbon-centered radicals ^+^R^•^ immediately reacting with molecular oxygen to form peroxyl radicals ^+^ROO^•^:^22^
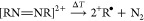
4
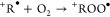
5^+^ROO^•^ further diffuses to the water/lipid interface and abstracts a H atom from MeLin (abbreviated as LH), providing effective initiation of chain reaction of MeLin peroxidation:^23,24^
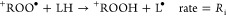
6
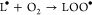
7
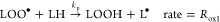
8The rate of peroxidation, *R*_ox_, is limited by reaction 8 (*k*_p_ < 10^2^ M^–1^ s^–1^ for linoleate),^25^ which is much slower than reaction 7 (*k*_8_ > 10^8^ M^–1^ s^–1^), and *R*_ox_ can be easily measured as the rate of oxygen consumption (−Δ[O_2_]/Δ*t*). A typical plot of oxygen uptake for the uninhibited peroxidation of LUVs (1:6:2 MeLin/DMPC/DMPG molar ratio) is presented in Figure 2 (control experiment, dashed line), together with the plots obtained in the presence of 1 μM 2,2,5,7,8-pentamethyl-6-hydroxychroman (PMHC, analogue of α-tocopherol), used as a reference chain-breaking antioxidant (solid line), and 5 μM DA, used as a model catecholamine (dash-dotted line). The results obtained in liposomes composed of DMPC and DMPG at a molar ratio of 3:1 are especially interesting, because this lipid system reflects the negative charge of the synaptic membrane (where even 20 mol % lipids are anionic).^26^

The results obtained in liposomes composed of DMPC, DMPC and DMPG at molar ratios of 1:1 and 1:3, and pure DMPG are presented in Figures S5–S8. For all studied systems, after injection of additives, the rate of peroxidation was significantly reduced to *R*_inh_, giving a clear induction period τ_ind_ (lag phase of peroxidation), as a consequence of peroxyl radical trapping by the antioxidant (PMHC or DA):^27^
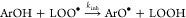
9After the antioxidant is consumed, the peroxidation rate increases from *R*_inh_ to *R*_ox2_ [postinhibited peroxidation (see Figure 2)]. The lengths of induction periods, τ_ind_, caused by PMHC or DA, were calculated as the integral:^28^
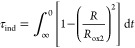
10where *R* is the rate of peroxidation after the addition of the antioxidant (initially *R* = *R*_inh_, but gradually increasing to reach *R*_ox2_). The values of parameters *R*_ox1_, *R*_inh_, τ_ind_, and *R*_ox2_ are listed in Table S6. Values of *k*_inh_ were calculated from the linear expression:^23^
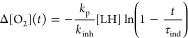
11where Δ[O_2_](*t*) is the oxygen uptake measured at time intervals *t* (within the induction period, *t* < τ_ind_), *k*_p_ is the rate constant of propagation (reaction 8),^29^ and [LH] is the concentration of MeLin. Values of *k*_inh_ are listed in Table 2. Values of the rate of initiation *R*_i_ were determined by the method proposed by Hammond et al.^30^ using PMHC as a model antioxidant:
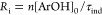
12where *n* is a stoichiometric factor, i.e., the number of LOO^•^’s scavenged per molecule of antioxidant (*n* = 2.0 for PMHC). Knowing the initiation rates and the values of τ_ind_ caused by DA, we determined the stoichiometric factor *n* for DA (values listed in Table 2).

**Table 2:** Kinetic Parameters Determined for the Peroxidation of 2.74 mM MeLin in DMPC/DMPG LUVs Inhibited by 1 μM PMHC or 5 μM DA^a^

	1 μM PMHC			5 μM DA		
model	τ_ind_ (min)	*R*_i_ (×10^9^ M s^–1^)	*k*_inh_ (×10^–4^ M^–1^ s^–1^)	τ_ind_ (min)	*k*_inh_ (×10^–3^ M^–1^ s^–1^)	*n*
micelles^b^	7.3 ± 0.5	4.6 ± 0.3	2.0 ± 0.2	no antioxidant activity		
LUVs^c^ (*X*_DMPG_ = 0.00)	9.6 ± 0.8	3.5 ± 0.3	1.3 ± 0.2	57.0 ± 5.4	1.6 ± 0.1	2.4 ± 0.2
LUVs^c^ (*X*_DMPG_ = 0.25)	6.0 ± 0.4	5.6 ± 0.3	1.9 ± 0.5	55.7 ± 2.4	1.4 ± 0.0	3.7 ± 0.2
LUVs^c^ (*X*_DMPG_ = 0.50)	5.9 ± 0.1	5.6 ± 0.1	1.7 ± 0.3	51.2 ± 4.1	1.3 ± 0.1	3.4 ± 0.3
LUVs^c^ (*X*_DMPG_ = 0.75)	5.9 ± 0.2	5.6 ± 0.2	2.3 ± 0.3	54.9 ± 7.7	1.3 ± 0.2	3.7 ± 0.5
LUVs^c^ (*X*_DMPG_ = 1.00)	5.3 ± 0.5	6.4 ± 0.7	1.4 ± 0.2	53.3 ± 4.5	1.2 ± 0.2	4.1 ± 0.3

a Length of the induction period (τ_ind_), rate of initiation (*R*_i_), inhibition rate constant (*k*_inh_), and stoichiometric factor (*n*). Experiments were performed at 310 K and pH 7.0 with ABAP as an initiator. All numbers represent the average values obtained from a series of measurements with calculated standard deviations.

b Kinetic parameters obtained for 2.74 mM MeLin peroxidation in Triton X-100 micelles were added for comparison.

c LUVs consisted of DMPC and DMPG, with *X*_DMPG_ being the molar fraction of DMPG in phospholipids [*n*_DMPG_/(*n*_DMPG_ + *n*_DMPC_)].

Table 3 presents two parameters, kinetic chain length of propagation (*v*) and efficiency of suppression (*eff*), derived for uninhibited, inhibited, and postinhibited peroxidation. The kinetic chain length of propagation *v*, defined in footnote *a* to Table 3, expresses the number of lipid peroxyl radicals formed per one initiating radical (i.e., the number of propagation cycles, reactions 7 and 8, triggered by one initiating radical). The efficiency of suppression quantifies the impact of the antioxidant added to the system on the propagation of lipid peroxidation indicating how many times the rate of peroxidation is reduced in the presence of an antioxidant compared to the rate of uninhibited peroxidation (see footnote *b* in Table 3).

**Table 3:** Kinetic Parameters^a^,^b^ Determined for Peroxidation of MeLin in DMPC/DMPG LUVs without Added Phenols or Inhibited by 1 μM PMHC or 5 μM DA^c^

	no additive	1 μM PMHC				5 μM DA			
model	*v*_ox1_^a^	*v*_inh_^a^	eff_inh_^b^	*v*_ox2_^a^	eff_ox2_^b^	*v*_inh_^a^	eff_inh_^b^	*v*_ox2_^a^	eff_ox2_^b^
micelles^d^	92.5 ± 7.3	4.6 ± 0.3	20.2 ± 0.9	57.2 ± 3.4	1.6 ± 0.1	no antioxidant activity			
LUVs^e^ (*X*_DMPG_ = 0.00)	16.8 ± 1.4	3.7 ± 0.4	4.3 ± 0.5	18.3 ± 2.5	0.9 ± 0.1	3.7 ± 0.2	4.7 ± 0.3	14.2 ± 0.9	1.2 ± 0.1
LUVs^e^ (*X*_DMPG_ = 0.25)	14.1 ± 1.1	2.1 ± 0.2	6.6 ± 0.5	16.1 ± 0.9	0.9 ± 0.1	3.6 ± 0.5	4.0 ± 0.6	14.2 ± 1.3	1.0 ± 0.1
LUVs^e^ (*X*_DMPG_ = 0.50)	13.6 ± 0.3	2.5 ± 0.3	6.7 ± 0.2	16.3 ± 0.5	0.9 ± 0.1	4.0 ± 0.3	3.6 ± 0.3	12.2 ± 1.0	1.2 ± 0.1
LUVs^e^ (*X*_DMPG_ = 0.75)	13.0 ± 1.3	2.4 ± 0.4	5.5 ± 0.9	13.8 ± 1.6	1.0 ± 0.1	4.1 ± 0.4	3.3 ± 0.3	11.7 ± 1.3	1.2 ± 0.1
LUVs^e^ (*X*_DMPG_ = 1.00)	10.1 ± 0.6	2.5 ± 0.5	4.3 ± 0.9	12.1 ± 1.1	0.8 ± 0.1	3.4 ± 0.2	3.4 ± 0.2	6.0 ± 0.5	1.9 ± 0.2

a Kinetic chain lengths for uninhibited (*v*_ox1_), inhibited (*v*_inh_), and postinhibited (*v*_ox2_) peroxidation (i.e., *v*_ox1_ = *R*_ox1_/*R*_i_, *v*_inh_ = *R*_inh_/*R*_i_, and *v*_ox2_ = *R*_ox2_/*R*_i_).

b Efficiencies of suppressing peroxidation calculated for the induction period (eff_inh_ = *R*_ox1_/*R*_inh_) and for the postinhibited process (eff_ox2_ = *R*_ox1_/*R*_ox2_).

c Experiments were performed at 310 K and pH 7.0 with ABAP as an initiator. All numbers represent the average values obtained from a series of measurements with calculated standard deviations.

d Kinetic parameters obtained for 2.74 mM MeLin peroxidation in Triton X-100 micelles were added for comparison.

e LUVs consisted of DMPC and DMPG, with *X*_DMPG_ being the molar fraction of DMPG in phospholipids, see footnote *c* in Table 2.

## Discussion

The progress of neurodegenerative diseases is associated with oxidative stress,^31^ and many attempts to develop relevant antioxidant therapies based on molecules originally present in the brain or able to diffuse through the blood-brain barrier (BBB) after systematic administration have been made.^32^ Here, we evaluated the potential antioxidant activity of three catecholamine neurotransmitters [dopamine (DA), adrenaline (ADR), and noradrenaline (NOR)] and the activity of l-3,4-dihydroxyphenylalanine (L-DOPA, a precursor of catecholamines, that effectively crosses the BBB and is already used in the clinical treatment of PD^33^).

The results from cell lines indicate antioxidant and cytoprotective activities of catecholamines,^5^ but the mechanism of their action remains unclear. Catecholamines, like other mono- and polyhydroxyphenols, react with free radicals via several mechanisms, depending on the properties of the reactants and on the reaction microenvironment (including solvent effects). Herein, we verified the hypothesis that catecholamines react via a SPLET mechanism in an aqueous environment by compiling studies on catecholamine acidity (and the related protonation status of their phenol groups) with kinetic studies of their reaction with model dpph^**•**^ in a homogeneous water/methanol system [1:1 (v:v)] at pH 5.5 and 7.4. Afterward, we evaluated the ability of DA (that has the highest radical trapping activity toward dpph^**•**^) to suppress lipid peroxidation in liposomes.

The ability of catecholamines to reduce free radicals can be predicted on the basis of their O–H bond strength, oxidation–reduction potentials, or relative parameters such as the HOMO/LUMO difference and the energy of stabilization of the radical formed after oxidation (regardless if it is a one-step or multistep process); for the values of some of the parameters, see Table 4. For example, analysis of BDE values indicates that the catechol moiety is crucial for the ability of catecholamines to trap the radicals. N–H bonds in DA, ADR, and NOR are much stronger (ΔBDE = 10–25 kcal/mol)^11^ than O–H bonds (BDE_O–H_ = 78.4–79.1 kcal/mol). For all catecholamines apart from DA, the O–H bond in the *para* position with respect to alkyl chain is the weakest (see footnote *b* in Table 4). The dominating reactivity of catechol hydroxyl was confirmed in all experimental and computational results accessible in the literature.

**Table 4:** Parameters Describing the Potential Antiradical Ability of Catecholamines^a^

	BDE_O–H_^b^	*D*_HT_^c^	HOMO^d^	*E*°′_(H_2_O)_^e^	*E*°′_(MeCN)_^f^
L-DOPA	79.1 (*81.3*)	–0.50	–8.61	0.308^34^–0.44^g^	1.52
DA	78.4 (*80.8*)	–0.69	–8.53	0.370^h^^,^^35^–0.42^34^	1.29
NOR	78.6 (*81.3*)	0.41	–8.76	0.384^36^	1.52
ADR	78.9 (*81.0*)	0	–8.68	0.372^36^	1.50

a O–H bond dissociation enthalpies (BDE_O–H_, in kilocalories per mole), HOMO energies (in electronvolts), energy difference between the phenoxyl radical and its parent catecholamine *D*_HT_ (in kilocalories per mole), two-electron reduction potentials in water [*E*°′_(H_2_O)_ vs NHE, in volts)], and oxidation potentials in acetonitrile [*E*°′_(MeCN)_ vs HNE, in volts)].

b Data calculated in benzene (and methanol, in italics).^11^ All listed values represent the weakest O–H bond in the *para* position, but exceptionally, for DA in benzene the O–H bond in the *para* hydroxyl is stronger (+0.9 kcal/mol) than the O–H bond in the *meta* position. In the same work, the BDE_O–H_ calculated for unsubstituted catechol was 79.8 kcal/mol in benzene and 82.2 kcal/mol in methanol.

c With respect to adrenaline, for which *D*_HT_ = 398.1 kcal mol^–1^. Data from ref (9c).

d Calculated by Ohkubo et al.^9c^ for catecholamines as ammonium cations. HOMO energies for neutral compounds are reported by Dimić et al.:^11^ −0.290 eV (for DA), −0.294 eV (for ADR and NOR), and −0.293 eV (L-DOPA).

e For the two-electron, two-proton (−2e/–2H^+^) oxidation potential. All values measured or recalculated vs NHE, unless otherwise stated.

f In acetonitrile, measured vs Ag/AgNO_3_ (0.01M)^9b^ and recalculated for NHE.

g Calculated for two-proton, two-electron reduction of L-DOPA to dopaquinone at pH 7.4 from the equation *E*°′_pH_ = *E*° – 0.059 × pH, where *E*° is a formal redox potential (0.745 V vs NHE at pH 0).^37^ The same calculations for pH 5.5 gave an *E*°′_5.5_ of 0.42 V.

h At pH 7.0, in agreement with values of 0.405 V^38^ and 0.40 V.^39^ The redox potential for nondeprotonated DA is 0.752 V^40^ or 0.801 V^41^ at pH 0, 0.612 V^41^ at pH 3.2, and 0.56 V at pH 4.5.^42^ The standard potential value (*E*°) for two-electron, two-proton (−2e/–2H^+^) reduction of DA quinone to DA was described as^40^*E*°′ = −47.93 × pH + 558.4 mV (vs Ag/AgCl, 3 M KCl) giving, after recalculation into NHE, *E*° = 0.75 V at pH 0, *E*°′_5.5_ = 0.491 V, and *E*°′_7.4_ = 0.40 V.

From comparison of the values of BDE_O–H_, one can predict that DA should be the most active in reducing free radicals (due to its lowest BDE_O–H_) in contrast to the least active L-DOPA. A slightly different order can be derived from values of the energy difference between a phenoxyl radical and its parent catecholamine, *D*_HT_, calculated by Ohkubo;^9c^ however, this parameter also shows that DA is the most reactive catecholamine (because the semiquinone radical formed from DA has the lowest energy within the series of catecholamines). The value of the third parameter, HOMO energy, also indicates that DA is a better electron donor than three other compounds [regardless of whether the amino group is protonated (see footnote *d* in Table 4)].

Two last columns of Table 4 list the parameters that are directly dependent on pH. The redox potentials of catecholamines in water at pH 7–7.4 (the third column of Table 4) are in good agreement with redox potentials of catechols (∼0.4 V).^43^ For catechols, electrochemically reversible voltammograms follow the Nernstian shift of −0.059 (*m*/*n*) V per pH unit (with *m*/*n* = 1 for the overall two-proton, two-electron process). The same electrochemical behavior is observed for catecholamines, but the process is irreversible.^37,43^ Reduction of DA quinone to dopamine is described by an *E*° of 0.75 V corresponding to a two-proton, two-electron process [at a strongly acidic pH (see footnote *h* of Table 3)],^44^ and thermodynamic cycles reveal the inverted order of one-electron redox potentials (+1.08 V for DA → DA semiquinone radical and 2-fold lower, +0.428 V, for DA semiquinone radical → DA),^45^ which justifies the problems with separation of both steps.^42,46^ Interestingly, a Pourbaix diagram for the overall two-proton, two-electron process is a straight line within the pH range of 0–10 whereas the component steps (one-electron processes) have inflections in the slopes at pH 4–6.^42^ Although we did not find the formal redox potentials for all four catecholamines determined within one experimental series in water at pH ∼7.0, data presented in the third column of Table 4 demonstrate very similar values of redox potentials for all catecholamines. However, different experimental conditions and experimental errors do not allow us to definitely state that one catecholamine is a better reducer than another. The results for the whole series of catecholamines are accessible in acetonitrile, and described by the authors as one-electron oxidation potentials (see the last column of Table 4),^9b^ indicating that DA is a stronger reducer than other catecholamines, in agreement with its highest HOMO level^9c^ and the smallest *D*_HT_ value.

From a thermodynamic point of view, the parameters such as BDE, redox potential, and acidity are correlated within the series of the same class of compounds.^47^ However, the mechanism of the reaction depends strongly on the polarity and solubility of a compound and on the environment; therefore, the parameters such as BDE, which are helpful for a fast, rough prediction of the antiradical activity of compounds embarked in a bulk lipid or the lipid core of biomembranes (bilayers), are less useful for prediction of the activity in a more polar environment like water or a lipid/water interface.

### Acidity Constants for Catecholamines

p*K*_a_ values obtained by us by spectrophotometric titration for DA (8.37, 10.25, and 12.49) are compared in Table S1 with the acidity constants for catecholamines, reported by others, that were selected as the most representative, frequently referenced, and generally accepted in the literature. Experimental p*K*_a1_ values for DA (Table S1) range from 8.37 to 9.06. p*K*_a2_ values are at least one unit higher than p*K*_a1_ values (ranging from 9.95 to 10.60), while the third deprotonation occurs at pH >12, which is too close to the self-ionization of water p*K*_w_ to be reliably determined by a spectrophotometric titration.^48^ Moreover, even traces of oxygen in such an alkaline solution can lead to substantial DA oxidation and, consequently, to the contamination of the sample by products of DA oxidation.^19a^ We suppose that a small shift in our experimentally determined p*K*_a1_ and p*K*_a2_ as compared to the literature values is the effect of a solvent (50% methanol in water, with the pH calibrated for this system) that is slightly different from those in other reports.

**Scheme 1:**
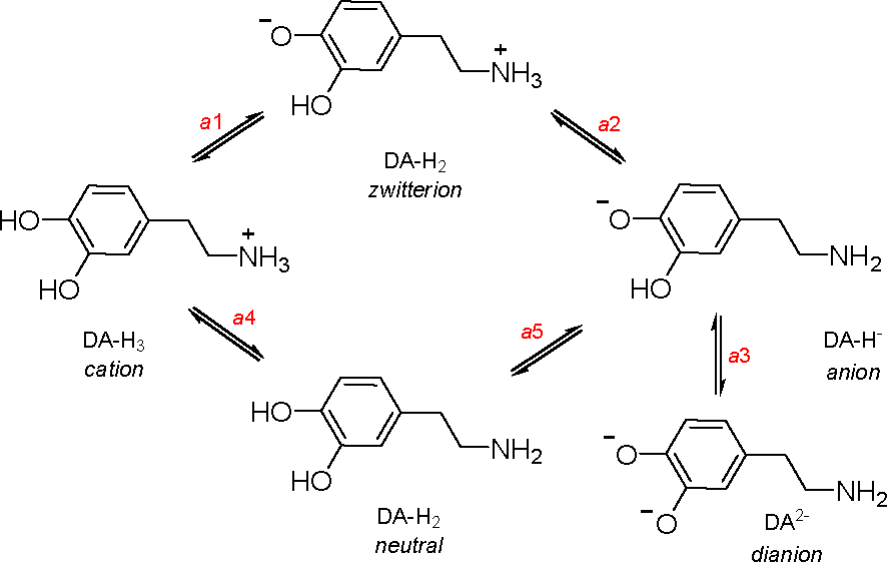
Possible Forms of DA during Deprotonation

By comparing p*K*_a_ values for DA determined in this study with the values reported for catechol (9.25) and ethanolamine, 2-phenylethylamine, and ethylamine [9.52, 9.89, and 10.68, respectively (see Table S1)], we assigned p*K*_a1_ = 8.37 and p*K*_a3_ = 12.49 to deprotonation of two phenolic groups of dopamine and p*K*_a2_ = 10.25 to deprotonation of the alkylammonium cation (see path a1 ⇆ a2 ⇆ a3 in Scheme 1). However, the p*K*_a_ values for the model compounds, catechol and amines, are too close to each other to definitely reach a conclusion about the order of dissociation, and some works questioned the dissociation scheme of DA and other catecholamines, with an alternative hypothesis that the ammonium cation is more acidic than the phenolic hydroxyl and dissociates as the first one (see pathway a4-a5 in Scheme 1).^49^

We hoped that a brief survey of the accessible reports about the acidity of catecholamines would be helpful for a correct interpretation of this problem; however, we discovered that the discussion initiated 50 years ago is still vivid (!), with the hypothesis of a stronger acidity of phenolic hydroxyl supported by ^1^H NMR experiments^50^ versus the arguments for superior deprotonation of the ammonium group at physiological pH, supported with ^13^C NMR and *ab initio* calculations.^51^ Already 40 years ago, Kiss and Gergely^48^ suggested (on the basis of earlier works by Martin^52^) that p*K*_a1_ and p*K*_a2_ cannot be assigned exclusively to deprotonation of the phenolic or ammonium group of DA but rather represent the superposition of these two processes, because there is a <10-fold difference in the values of microconstants, calculated separately for deprotonation of -OH and -NH_3_^+^ groups, resulting in a partial overlapping of deprotonation of these two moieties in DA. Thus, in the cascade of deprotonation equilibria a1 ⇆ a2 (zwitterion) ⇆ a3, the [zwitterion]:[anion] ratio is ∼10:1. A similar conclusion about overlapped deprotonation but with an additional indication that that first proton comes from OH being at a position *para* to the ethylammonium group was drawn by Gerard et al.^53^ and confirmed by theoretical calculations.^54^

Assuming that experimentally determined p*K*_a1_–p*K*_a3_ describe processes a1–a3, respectively, we plotted the dissociation diagram for DA presented in Figure S1 [diagrams for L-DOPA, NOR, and ADR (Figures S2–S4, respectively) were constructed from literature p*K*_a_ values]. Taking into account the fact that the [zwitterion]:[anion] ratio will be 10:1,^48^ the molar fraction of phenolate at pH 7.4 will be approximately the same because the second deprotonation step does not produce phenolate anions. However, if the order of deprotonation is reversed (a3-a4 in Scheme 1), the amount of phenolate anions will be ∼10 times smaller and at pH 7.4 the [zwitterion]:[neutral] ratio will be ≈0.1 with the zwitterion being the only form with a deprotonated phenol group.^51a^

The problem of whether DA, NOR, or ADR is in a neutral, monocationic, or zwitterionic form is of great importance for their interactions with adrenergic and dopaminergic receptors.^16^ However, in our kinetic studies in a methanol/water solution, the most important is the ionization of the catechol moiety, which plays a crucial role in the redox properties of catecholamines and their reaction with dpph^•^ (proceeding via HAT or ET mechanisms).

### Kinetics of the Reaction with dpph^•^

Taking into account the pH-dependent redox properties of catecholamines, we decided to use pH 7.4 as the standard physiological pH and pH 5.5 as the lower limit in brain tissues with chronic disease^44^ in our studies of the reaction of catecholamine with dpph^•^. Both pH values are within the Nernstian behavior of the studied catecholamines, and as one can predict, the increasing pH should be accompanied by an improving ability to reduce dpph^•^. To avoid the participation of further oxidation products in the overall reaction kinetics, we studied the very initial rates of reaction (the deadline for mixing was 8 ms; then, the kinetic traces were recorded within <0.5 s). Bimolecular rate constants determined at pH 5.5 (*k*^pH5.5^) are within the range of 290–1200 M^–1^ s^–1^ (see Table 1), being in reasonable agreement (within an order of magnitude) with *k*^S^ determined in neat methanol for reactions of dpph^•^ with unsubstituted catechol (*k*^MeOH^ = 300 M^–1^ s^–1^)^15^ and with phenols bearing a catechol moiety and a carboxyl group (which suppresses the ionization of phenol hydroxyls), like caffeic acid (*k*^MeOH^ ≈ 1200 M^–1^ s^–1^^55^ or 1100 M^–1^ s^–1^).^15,56^ The presence of a carboxyl group in caffeic acid has an effect comparable to that of a buffered system (pH 5.5) or to the presence of 10 mM acetic acid, and values of *k*^pH5.5^ listed in Table 1 are also in agreement with bimolecular rate constants determined in methanol containing 10 mM acetic acid for the reaction of dpph^•^ with 7,8-dihydroxyflavone (strongly acidic catechol moiety in ring A, p*K*_a_ = 7.4, and *k*^10 mMAcOH^ = 1500 M^–1^ s^–1^),^14b^ and for a noncatecholic flavone, morin (p*K*_a_ = 5.2, and *k*^10 mMAcOH^ = 750 M^–1^ s^–1^).^14b^ In both flavonoids, the most acidic hydroxyl group reacts as a phenolate anion. The concentration of H^+^ in methanol produced by 10 mM acetic acid having a p*K*_a_ of 9.63 in methanol^57^ is (0.01 M × *K*_a_)^0.5^ = 1.5 × 10^–6^ M, giving p[H^+^] 5.8, which corresponds to pH 5.5 in water or a water/methanol mixture.

For all catecholamines, an enormous acceleration of the reaction with dpph^**•**^ is observed when passing from pH 5.5 to 7.4. For NOR, the increase in *k*^S^ is ∼50 times, while for the three other catecholamines, the ratios (*k*^pH7.4^/*k*^pH5.5^) reach 130–160 and such a 2 order of magnitude increase in *k*^s^ follows the increase in the degree of deprotonation (α in Table 1) calculated from p*K*_a_ values. The general term “degree of deprotonation” used in Table 1 was used to avoid the speculation about whether α represents phenolate anions (if p*K*_a1_ is assigned to the OH group) or the degree of deprotonation of the ammonium cation to form amine. This latter case does not exclude the possibility of formation of phenolate anions, but their concentration will be 10–50 times smaller, as could be calculated from the 1–1.5 unit differences between p*K*_a1_ and p*K*_a2_. If the microconstants for acidic dissociation discussed in several papers mentioned above are taken into account, the real contribution of phenolate anions to the overall degree of dissociation will be at least 1/10 as stated by Kiss and Gergely.^48^ Regardless of the pattern of dissociation, the 2 order of magnitude difference in *k*^S^ clearly correlates with the 2 unit difference in pH and the 2 order of magnitude difference in the concentration of phenolate anions. In our opinion, this is one of the best pieces of quantitative evidence of the participation of the SPLET mechanism for catecholamines reacting with dpph^•^ in a water/methanol mixture. The question of whether this effect could be extended to the reactions of catecholamines with peroxyl radicals arises. The increase in the inhibition rate constants (reaction 9) with an increase in pH has already been reported for a simple catechol (p*K*_a1_ = 9.3) reacting with 2-tetrahydrofuranyl-peroxyl radicals in a water/THF mixture.^58^ The increase in pH from 2.1 to 7.4 resulted in an only 2-fold increase in *k*_inh_, which was followed by a nearly 100-fold increase when the pH was changed from 7.4 to 12. Such an enhancement of inhibition was explained by two nonexclusive mechanisms: a fast ET from monodeprotonated catechol (SPLET mechanism) and possible acceleration of HAT from the remaining OH group being weaker (with smaller BDE_O–H_) when a strong electron-donating phenoxide −O^–^ group is present at the *ortho* position. Although both mechanisms can act in parallel for peroxyl radicals, the reaction of anionic forms of catecholamines with dpph^•^ should proceed mainly via ET because dpph^•^ are ∼3 orders of magnitude less reactive than peroxyl radicalsin the abstraction of the hydrogen atom from ArOH,^7a^ but dpph^•^ are much more electron deficient than LOO^•^.

At both examined pH values, DA proves to have the highest reactivity toward dpph^•^ among catecholamines, as expressed by its largest value of *k*^S^. DA (anion) reacts with dpph^•^ so fast that *k*^pH7.4^ = 1.7 × 10^5^ M^–1^ s^–1^ becomes comparable to *k* = 2.2 × 10^5^ M^–1^ s^–1^ theoretically predicted by Iuga et al.^10^ for DA reacting with HOO^•^ in an aqueous environment. However, the authors suggested (on the basis of the assumption that p*K*_a1_ is assigned to dissociation of the ammonium cation and neglecting the presence of any traces of phenolate in the water system) that the initial reaction of DA with HOO^•^ is a two-step process with single-electron transfer (SET) followed by deprotonation of the highly acidic radical cation.^10^ Such a mechanism would be possible in less polar solvents and in the presence of a metal cation,^59^ but our results in a water/methanol system (with a pH-dependent rate for the reaction of catecholamine with dpph^•^) clearly demonstrate that the presence of phenolate anions cannot be neglected in the overall kinetics.

*k*^pH7.4^ exceeds the rate constant of 1.37 × 10^4^ M^–1^ s^–1^ determined for dpph^•^ reacting with catechin in a methanol/water mixture (6:4) but agrees with *k* = 6.1 × 10^5^ M^–1^ s^–1^ obtained after the extrapolation to neat water and interpreted as the rate constant for ET from the catechin anion to dpph^•^.^60,61^ With the assumption that the α parameters listed in Table 1 represent the anionic fractions of DA (in percent), the overall rate constant *k*^pH7.4^ can be converted into *k*^ET^ ≈ (100/α)*k*^pH7.4^ ≈ 2 × 10^6^ M^–1^ s^–1^, in full agreement with the theoretical *k*^ET^ = 3.3 × 10^6^ M^–1^ s^–1^ calculated for DA anions reacting with dpph^•^ (in the gas phase).^11^

The highest reactivity of DA among the series of four catecholamines agrees with the thermodynamic parameters listed in Table 4 and agrees with observations made by other researchers. Kawashima et al.^9b^ correlated the highest reactivity of DA among catecholamines with its lowest oxidation potential *E*_ox_ (see Table 4) and noticed that for the reaction with galvinoxyl radical in acetonitrile, DA was the only neurotransmitter that enhanced the reactivity in the presence of Mg^2+^ cations, indicating the MCET mechanism, whereas other (L-DOPA, ADR, and NOR) reacted via pure HAT. Our data confirm that DA is the most active; however, in a buffered methanol/water mixture, all four catecholamines (their anions) are excellent reducing agents. The highest reactivity of DA corresponds to almost all of the parameters listed in Table 4, and additional enhancement of reactivity is achieved due to its stronger acidity resulting in a higher degree of dissociation degree at a given pH than for other catecholamines.

### Experiments in Model Lipid Systems

The ability of DA and L-DOPA to inhibit peroxidation in micellar systems has already been verified by us for MeLin/Triton X-100 micelles over an extended pH range.^18^ To our surprise, neither DA nor L-DOPA was able to sufficiently break the kinetic chain of peroxidation to produce the inhibition period (lag phase), although both catecholamines retarded peroxidation at acidic and neutral pH. A similar behavior was reported for some phenolic acids with the catechol moiety in PC liposomes.^62^ We suggested that the concentration of DA and L-DOPA in the lipid micellar phase was too low to effectively suppress the intramicellar peroxidation. Instead, both catecholamines were mainly localized in water and trapped some fraction of water-soluble initiating radicals. Our explanation agrees with values of the octanol/water partition coefficients (log *P* values of −0.99 for DA and −2.38 for L-DOPA),^63^ indicating the preferential aqueous location for both catecholamines, and also agrees with the early works on the ability of catecholamines to permeate model lipid bilayers (dioleoyl-PC/cholesterol) showing no transport of catecholamines through the membrane in the absence of a negatively charged ionophore or an ion pairing agent.^64^ However, the results of molecular dynamics simulations suggest that catecholamines should interact with membrane lipid headgroups via hydrogen bonds and electrostatic interactions.^17a^ At physiological pH, catecholamines occur predominantly as cations, with smaller fractions of zwitterions (DA, ADR, and NOR), or as zwitterions with a smaller fraction of the anionic form (L-DOPA), all having a positive charge on the ammonium group.^19a,52^ In our microcalorimetric study,^17b^ we demonstrated that positively charged ammonium groups of DA interact superficially with anions of phospholipid headgroups, and these interactions were stronger for zwitterionic 1,2-dimyristoyl-*sn*-glycero-3-phosphocholine (DMPC) enriched with anionic 1,2-dimyristoyl-*sn*-glycero-3-phosphoglycerol (DMPG).^17b^ These findings were confirmed by NMR technique^65^ and also by fluorescent probes;^66^ however, there is still a discussion about whether dopamine is adhered on the membrane surface^17b,65b^ or whether DA can penetrate the hydrophobic core of the membrane.^17a,65,66^ Thus, we decided to check the ability of dopamine to act as a chain-breaking antioxidant during MeLin oxidation in DMPC/DMPG liposomes with an increasingly negative surface charge. We selected DA as the catecholamine expected to have the highest radical trapping activity among catecholamines, as predicted on the basis of the values of the parameters listed in Table 4 and our results from studies with the model dpph^•^ radical.

### Uninhibited Peroxidation

Peroxidation of MeLin in liposomes in the absence of antioxidants proceeds at a constant rate *R*_ox1_, which is ∼5 times smaller than the *R*_ox1_ determined earlier by us for MeLin peroxidation in Triton X-100 micelles (42.2 × 10^–8^ M s^–1^, see Table S6).^18^ The slowest uninhibited peroxidation was detected for liposomes composed of pure DMPC (60 nM s^–1^), while for liposomes enriched with DMPG, *R*_ox1_ slightly increased, but was independent on the amount of DMPG, with an average value of 77 ± 4 nM s^–1^ calculated for LUVs containing 25–100 mol % DMPG. The differences in the rates of peroxidation proceeding in micelles versus bilayers as well as in neutral versus charged bilayers can arise either from the different susceptibilities of the studied systems to initiation (reaction 6) or differences in propagation of peroxidation within the lipid phase (reaction 8). In our experiments peroxidation was initiated by ABAP, which slowly decomposes at 310 K providing a constant radical flux, indispensable in quantitative kinetic studies. ABAP decomposes to positively charged carbon-centered radicals ^+^R^•^, which are immediately converted to peroxyl radicals ^+^ROO^•^, that attack the membrane. *R*_i_ is ∼40% lower for liposomes composed of pure DMPC than for liposomes enriched with DMPG (independent of DMPG content) (see Table 2). It can be explained by the electrostatic attraction of the positively charged initiating radicals toward the membrane surface. To extract information about differences in propagation processes in the lipid phase, we calculated the values of kinetic chain lengths of propagation *v*. Interestingly, the largest *v* of 17 was obtained for liposomes composed of pure DMPC and *v* decreased with membrane charge to only 10 peroxyls per initiating radical formed in DMPG LUVs. Thus, although the initiation is favored for negatively charged liposomes, the propagation processes are slower and less effective, probably because of the repulsion between negatively charged lipids and the less ordered structure of DMPC/DMPG and DMPG membranes as compared to pure DMPC bilayers (indicated by the smaller area per lipid in DMPC than in DMPG).^67^

The observed differences in the propagation of lipid peroxidation in micelles versus a bilayer can arise from the different mobility of MeLin, resulting from the different stability of both systems (dynamic micelles with short half-lives between monomers and aggregates vs membranes composed of relatively static aggregates),^68^ and the high microviscosity of membranes compared to that of micelles.^69^ The rate of uninhibited peroxidation is proportional to the lipid concentration in the nonpolar phase. On the basis of a comparison of MeLin:surfactant and MeLin:PC molar ratios, we can roughly assume that MeLin is more concentrated in Triton X-100 micelles than in the bilayer, what can also account for faster oxidation in micelles.^70^

### Antioxidant Properties of PMHC in Liposomes

PMHC behaves as a chain-breaking antioxidant in the DMPC/DMPG system as indicated by quite pronounced induction periods recorded for all evaluated liposomal systems (Figure 2 and Figures S5–S8). There is no clear relationship between the value of the bimolecular rate constant of inhibition for the reaction of PMHC with peroxyls, *k*_inh_, and the negative charge of liposomes, with the average value for *k*_inh_ of (1.7 ± 0.4) × 10^4^ M^–1^ s^–1^. Thus, we suggest that the membrane surface charge does not affect the chain breaking activity of PMHC, because, as a hydrophobic molecule, PMHC penetrates the membrane core and its interactions with the membrane do not depend on electrostatics. The obtained value of *k*_inh_ in DMPC/DMPG liposomes perfectly agrees with *k*_inh_ = 1.78 × 10^4^ M^–1^ s^–1^ determined for PMHC suppressing the peroxidation of a dilinoleoylphosphatidylcholine (DLPC) membrane.^23^ Moreover, it is very close to the *k*_inh_ describing the activity of PMHC in Triton X-100 micelles (*k*_inh_ = 2.0 × 10^4^ M^–1^ s^–1^).^18^ Thus, despite the much lower sensitivity of the liposomal system to peroxidation, model antioxidant PMHC gave coherent results in DMPC/DMPG liposomes and Triton X-100 micelles.

However, the magnitude of suppression of lipid peroxidation by PMHC (expressed by the parameter *eff*_inh_) is significantly lower in the bilayer than in micelles. Namely, PMHC causes an ∼5.5-fold decrease in the rate of peroxidation in DMPC/DMPG LUVs (again, with no clear dependence on the liposome charge) as compared to the 20-fold decrease in Triton X-100 micelles. After the end of τ_ind_, MeLin peroxidation in LUVs proceeds at a rate comparable to that of uninhibited peroxidation (i.e., *eff*_ox2_ ≈ 1.0), indicating the total consumption of PMHC in the system. Such behavior differs from the observations described for Triton X-100 micelles, where after the end of the induction period the peroxidation rate was still lower than that of the uninhibited process.

### Antioxidant Properties of DA in Liposomes

In contrast to results for the micellar system, where DA only retarded lipid peroxidation, in DMPC/DMPG liposomes the antioxidant action of DA is manifested by a clear induction period (see Figures S5–S8 and Table 3 and Table S6), indicating a direct reaction of DA with peroxyl radicals. The induction period caused by DA (applied at a concentration 5 times higher than that of PMHC) is nearly 10 times longer than the τ_ind_ determined for PMHC, but its length does not depend on the charge of the membrane (i.e., the fraction of DMPG), having an average value of 54.4 ± 2.2 min. The rate constant for a bimolecular reaction of DA with peroxyl radicals is 1 order of magnitude lower than *k*_inh_ determined for PMHC in the bilayer system and is larger for liposomes composed of pure DMPC (*k*_inh_ = 1.6 × 10^3^ M^–1^ s^–1^) than for mixed DMPC/DMPG LUVs, where the average value of *k*_inh_ is (1.3 ± 0.1) × 10^3^ M^–1^ s^–1^. Such a low value of *k*_inh_ classifies DA as a weak chain-breaking antioxidant, with *k*_inh_/*k*_p_ ≈ 10^2^ being the border value for suppression of a chain of lipid peroxidation. Interestingly, both parameters, τ_ind_ and *k*_inh_, do not depend on the fraction of DMPG in the membrane, although the strength of DA/membrane interactions increases with the membrane negative charge due to electrostatic forces.^17b^ Moreover, the *k*_inh_ is maximal for the neutral DMPC membrane, where electrostatic forces are negligible. Thus, it seems that a detectable antioxidant effect of DA in the liposomal system cannot be explained by only a simple attraction of DA to the membrane surface, resulting in a local increase in its concentration in the proximity of the membrane. It is rather the proper orientation of the molecule in the membrane that is crucial for the manifestation of the antioxidant activity. The hydrophobic component of interactions of dopamine with the DMPC/DMPG membrane was extracted from our microcalorimetric data^17b^ as the intrinsic partition coefficient, which is independent of the membrane charge. For pure DMPG liposomes, the hydrophobic effects are 10 times weaker than the electrostatic forces, but the ratio of electrostatic to hydrophobic interactions decreases for a decreasing fraction of DMPG in the DMPC/DMPG membrane, with hydrophobic effects being entirely responsible for interactions of DA with the neutral DMPC membrane. Thus, the hydrophobic interactions of DA allow penetration of the lipid bilayer, as already suggested by some researchers,^17a,65,66^ and the degree of this intercalation is enough to enable DA to act as a weak antioxidant. So far, the role of membrane interactions in antioxidant activity has been described for some nonsteroidal anti-inflammatory drugs from the oxicam family.^71^ Thus, the lack of antioxidant activity of DA in Triton X-100 micelles reported earlier by us can be explained by overly weak interactions between DA and non-ionic micelles. The second possible explanation of the different behavior of DA in liposomes and micelles is substantially faster propagation of lipid peroxidation in micelles than in liposomes, reaction 8 dominates over reaction 9, and the induction period is not manifested.

**Figure 3:**
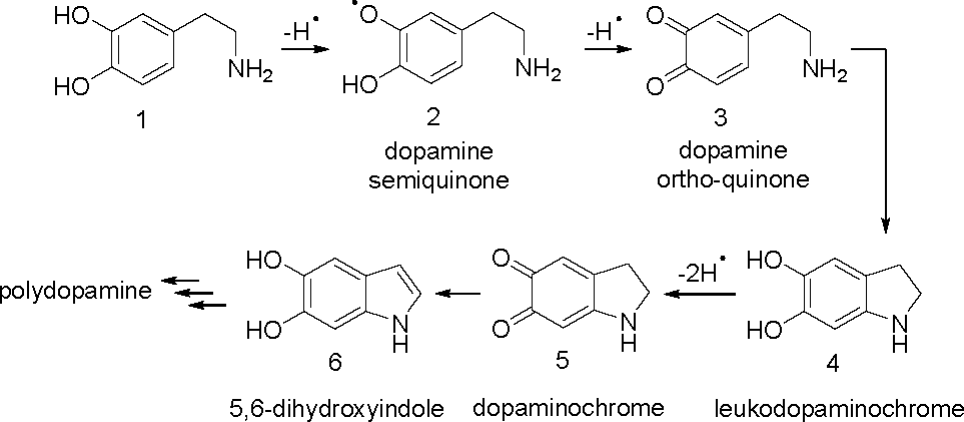
Oxidation of dopamine leading to polydopamine with the first steps including recovery of the catechol moiety.^42,44^ For the sake of simplicity, amino and hydroxyl groups are also presented in non-ionized forms, and oxidation is visualized as abstraction of a H atom, regardless of a one-step or multistep sequence.

The stoichiometric factor *n* of 2.4 ± 0.2 determined for DA in DMPC is exactly the same as that reported by Barclay^72^ for reaction of catechols with alkylperoxyl radicals in chlorobenzene and reflects the two-step oxidation of catechol to semiquinone, and subsequently to quinone.^73^ Barclay noticed a decrease of *n* to 1.1 in micellar systems (SDS, pH 7) and interpreted this effect as a partial oxidation of catechol in processes other than the chain-breaking action. Our results indicate that DA behaved in a manner different from that of simple catechols, and the *n* of 2.4 in DPMC increases to 3.7 ± 0.3 in DMPC/DMPG liposomes (see Table 2). Such a high value of *n* might be explained by the participation of not only two hydroxyl groups but also the amino group in further reactions of the oxidized quinone, with cyclization to products with a recovered catechol moiety (leucodopaminochrome, dopaminochrome, and dihydroxyindole)^9a,19b,42,44^ being the first steps of production of polydopamine (see Figure 3). We suppose that the negative charge of DMPG facilitates some of these processes, and, additionally, increases the concentration of the polymerizing species.

Although the hypothesis that OH groups are recovered due to nucleophilic attack of nitrogen on *o*-quinone and rearomatization (see Figure 3) seems to be the most probable and agrees with the mechanism of oxidative polymerization of dopamine under alkaline conditions, other mechanisms cannot be excluded. For example, the increase in the stoichiometric factors was also observed for cinnamic acid derivatives inhibiting autoxidation of tetrahydrofuran in water, which was attributed to rapid Michael type nucleophilic addition of water to *o*-quinone, and enolization (reaction 13); however, this process was facilitated by an electron-withdrawing group (EWG) present in the side chain.^74^
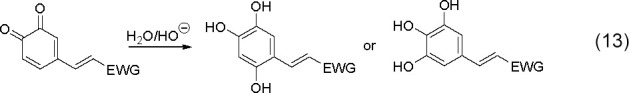
13An anonymous reviewer suggested another possible explanation for the recovery of hydroxyl groups, the reduction of *o*-quinone by hydroperoxyl radicals generated during autoxidation of the side chain of structure 3 (in Figure 3). Reduction of *o*-quinone to catechol by HOO^•^ is well-documented,^75^ but we exclude this mechanism in our lipid/water system because water-soluble hydroperoxyl radicals would immediately escape from the interphase into bulk water and, additionally, at neutral or alkaline pH almost all HOO^•^ radicals (p*K*_a_ = 4.8) will undergo a fast deprotonation to less reactive superoxide O_2_^•–^.

Despite the elongated time of activity, DA is slightly less efficient than PMHC (5 μM DA causes an ∼4-fold decrease in the peroxidation rate, with the largest value of *eff*_inh_ observed for pure DMPC liposomes, compared to an ∼5.5-fold decrease caused by 1 μM PMHC). After the induction period is finished, both compounds have a negligible impact on the peroxidation rate in DMPC and mixed DMPC/DMPG LUVs; however, in DMPG LUVs, DA causes a nearly 2-fold decrease in the rate of postinhibition peroxidation, in agreement with the hypothesis that a negatively charged microenvironment favors the formation of products (Figure 3) that can trap ROS.

In contrast to the peroxidation of methyl linoleate in micellar non-ionic systems (Triton X-100),^18^ data presented herein give the experimental evidence that DA can efficiently suppress lipid peroxidation in DMPC/DMPG LUVs, as model bilayers relevant to biological systems. This is consistent with results of the studies showing that DA protects brain homogenate against lipid peroxidation.^12^ However, the studies on brain homogenates were performed in the presence of metal ions, so it was difficult to evaluate whether DA acted as a chain-breaking antioxidant or as a preventive antioxidant (that forms complexes with metal ions). Metal binding as a main mechanism explaining the protective effect of DA against oxidative stress has already been suggested by some authors.^76^ In contrast, our results represent the experimental proof of the chain-breaking activity of DA in a model heterogeneous water/lipid system.

## Conclusions

We present quantitative data on the ability of four catecholamines (DA, L-DOPA, ADR, and NOR) to scavenge free radicals in an aqueous solution and in a phospholipid bilayer. In a water/methanol solution at a precisely controlled pH, catecholamines scavenge the model dpph^•^ radical in a fast and effective way. The large, 2 orders of magnitude, enhancement of their reactivity with an increase in pH from 5.5 to 7.4 provides the clear evidence that scavenging activity is correlated with deprotonation of hydroxyl groups, with participation of fast electron transfer from the phenolate anion to dpph^•^ (as predicted by the SPLET mechanism) in addition to much slower one-step HAT. The observed acceleration agrees with the parameters describing the pH-dependent redox potentials of catecholamines as well as with the thermodynamic descriptors of the stability of radicals formed after abstraction of a H atom from catecholamines (in a one- or two-step process). The experiments carried out in model lipid membranes demonstrated that dopamine efficiently breaks the chain of peroxidation of an unsaturated lipid dispersed in model phospholipid membranes assembled from zwitterionic DMPC and negatively charged DMPG (at different molar ratios). Interactions of DA with the phospholipid membrane and diffusion phenomena are important factors governing the radical trapping ability of this catecholamine, with a small increase in activity observed in negatively charged liposomes. The value of the stoichiometric factor, *n*, indicates that approximately four peroxyl radicals are trapped per molecule of DA in anionic membranes, exceeding the values for catechols in liposomal systems. Such a high value of *n* indicates that some products of oxidative modification of DA (such as leucoaminochrome and 5,6-dihydroxyindole) can also be responsible for trapping of peroxyl radicals.

This work clearly demonstrates the need to measure the antioxidant activity in various model systems to obtain a more complete picture of the potential protective activity of the compound against the free radicals. From the point of view of the resemblance to physiological conditions, data obtained in the bilayer system are the most convenient. Biological membranes are far more diverse in lipid composition, structure, and the presence of membrane proteins, other antioxidants, and prooxidants (including metal ions), which can dramatically change the antioxidant properties of catecholamines and their further fate when undergoing enzymatic and non-enzymatic reactions after reactions with ROS.^77^ Thus, the kinetic data presented here demonstrate the potential ability of a model catecholamine, dopamine, to trap peroxyl radicals in the lipid membrane.

## Experimental Section

### Materials

Dopamine hydrochloride (DA, 98.5%, powder, Sigma-Aldrich), l-3,4-dihydroxyphenylalanine (L-DOPA, 97%, powder, Sigma-Aldrich), (−)-adrenaline (ADR, >98%, powder, Sigma-Aldrich), l-noradrenaline (NOR, >98%, powder, Fluka), 2,2,5,7,8-pentamethyl-6-hydroxychroman (PMHC, 97%, powder, Sigma-Aldrich), 2,2'-diphenyl-1-picrylhydrazyl radical (dpph^•^, 95%, powder, Sigma-Aldrich), 2,2′-azobis(2-methylpropionamidine) dihydrochloride (ABAP, 97%, powder, Sigma-Aldrich), methyl linoleate (MeLin, 99%, liquid, Sigma-Aldrich), 1,2-dimyristoyl-*sn*-glycero-3-phosphocholine (DMPC, 99%, powder, Avanti Polar Lipids), 1,2-dimyristoyl-*sn*-glycero-3-phosphoglycerol (DMPG, 99%, powder, Avanti Polar Lipids), solvents, and buffer constituents were used without further purification.

### Determination of the Acidity Constants p*K*_a_ of Dopamine

Because of the high sensitivity of dopamine (DA) to oxidation, we modified the standard spectrophotometric titration method applied previously by us for flavonoids and prepared each pH sample separately, as in point by point analysis proposed by Sánchez-Rivera et al.^19a^ In this method, a fresh DA stock solution (concentration of 1.13 × 10^–2^ M) was prepared and kept under anaerobic conditions. For each spectrophotometric determination, a 100 μL aliquot of stock solution was added to a 10 mL sample of 10 mM H_3_PO_4_ previously titrated with KOH to achieve the desired pH value (the pH for each sample was determined by a precision pH meter with a combined pH glass electrode just before the addition of the DA stock solution). The samples containing added DA (final concentration of 1.13 × 10^–4^ M) were immediately transferred into a quartz cuvette, and an ultraviolet–visible (UV–vis) spectrum within the range of 200–600 nm was recorded with a UV–vis Cary 50 spectrometer. The experiments were performed under nitrogen to minimize the extent of dopamine oxidation. The spectra obtained for pH values ranging from 1.5 to 12 were processed with Datan version 3.1 (MultiD Analyses AB) to determine acidity constants.

### Kinetic Measurements of the Rate Constant for Reactions of Catecholamine with dpph^•^

The rates of reaction of dpph^•^ with catecholamines were monitored using the stopped-flow technique, and the rate constants were calculated as described previously^14^ in a number of neat organic solvents; however, in the work presented here, the methodology was applied for the water/methanol system. Solutions of dpph^•^, DA, L-DOPA, ADR, and NOR in a 1:1 (v:v) water/methanol mixture at pH 5.5 (acetate buffer consisting of 21.4 mM CH_3_COONa and 3.6 mM CH_3_COOH) and at pH 7.4 (Tris buffer consisting of 50 mM Tris and 42 mM HCl) were prepared immediately before the experiments, in deoxygenated (nitrogen-purged) solvents, and kept under nitrogen. Experiments were carried out at 296 ± 2 K. A series of measurements were performed at a fixed initial concentration of dpph^•^ (30 μM at pH 5.5 and 6 μM at pH 7.4) and varied initial concentrations of catecholamines, always in stoichiometric excess over [dpph^•^], up to 500 μM at pH 5.5 and up to 50 μM at pH 7.4 (see the Supporting Information). The decay of dpph^•^ reacting with catecholamines was followed at 517 nm on a model RX-2000 stopped-flow rapid mixing accessory (Applied Photophysics) coupled to a CARY 50 UV–vis spectrophotometer (equipped with a 150 W xenon lamp). For a series of different starting concentrations of catecholamines, [Ar(OH)_2_]_0_, the pseudo-first-order experimental rate constants [*k*_exp_ (inverse seconds)] for the reaction of catecholamine and dpph^•^ were determined and the second-order rate constants (i.e., bimolecular rate constants) [*k*^S^ (inverse molar seconds)] were calculated from the slopes of the straight line dependence of *k*_exp_ versus [Ar(OH)_2_]_0_. For each pH, at least two independent series of experiments were performed.

### Preparation of Liposomal Suspensions for Oxygen Uptake Measurements

Liposomal suspensions were prepared from methyl linoleate (MeLin), DMPC, and DMPG. Each time, the weighed amount of DMPC was dissolved in chloroform and DMPG in a chloroform/ethanol solution [1:1 (v:v)]. These organic stock solutions of lipids were mixed in appropriate proportions to give various DMPC:DMPG molar ratios, and MeLin was added with a microliter automatic pipet to give a molar ratio of phospholipids to MeLin of 8:1. Afterward, the lipid solution was transferred to a pear-shaped glass flask. Organic solvents were evaporated in a vacuum rotary evaporator, and the flask was kept overnight under vacuum to remove traces of solvents. Subsequently, the thin lipid film was suspended in an appropriate amount of warm 50 mM phosphate buffer (pH 7.0) by being intensively shaken (vortexing) at a temperature above the lipid phase transition to give multilamellar vesicles (MLVs) with final concentrations of lipids: 2.74 mM MeLin and 21.92 mM phospholipids (DMPC and DMPG). Suspensions of MLVs were extruded at least 21 times through polycarbonate membranes with a pore diameter of 100 nm in an Avanti Mini-Extruder to afford 100 nm LUVs.

### Oxygen Uptake Measurements of Antioxidant Activity of Dopamine and PMHC

The antioxidant activities of DA and PMHC (a reference chain-breaking antioxidant) were investigated by the oxygen uptake method with MeLin (entrapped in LUVs composed of DMPC and DMPG) used as a substrate for peroxidation. The procedure was the same as described previously for a sodium dodecyl sulfate micellar system^21b^ and a Triton X-100 micellar system.^18^ Two milliliters of a liposomal suspension was transferred into the glass vessel placed in a thermostated bath of a model 5300A Biological Oxygen Monitor (Yellow Springs Instruments) with a model 5304 Micro Adapter Kit (converting the chamber volume to 2 mL). The constant stirring of the sample was assured by a magnetic stirrer with a stirring speed 480 rpm. After aeration for 10 min, a Clark type polarographic oxygen probe immersed in a Teflon plunger was placed in the vessel and the oxygen content was continuously recorded. The electrode was calibrated for air-saturated (100%) and degassed (0%) liposomal dispersions. In a single run, the oxygen contents in two separate samples were measured and the third chamber was used to control the temperature with a thermocouple. The bath assembly was connected to a constant-temperature circulator providing a temperature-controlled environment with a temperature stability 310 ± 0.2 K. The access slot along one side of the plunger enabled the removal of gas bubbles from the sample as well as the injection of the initiator and antioxidants to the sample. MeLin peroxidation was initiated by ABAP added with a microliter syringe (0.5 M aqueous stock solution) to afford a final concentration of 10 mM ABAP in the vessel. For the control experiment, no inhibitor was added and the rate of uninhibited peroxidation, *R*_ox1_, was determined from the plot of oxygen uptake, Δ[O_2_], versus time, *t*. For experiments on peroxidation inhibited by DA or PMHC, after the initiator was added and a constant rate of oxygen uptake started, 4 μL of DA (in water) or 4 μL of PMHC (in ethanol) was injected and the rate of inhibited peroxidation, *R*_inh_, was measured during the induction period (lag phase of peroxidation), together with the length of the induction period, τ_ind_ (determined by the method of Roginsky^28^), and the rate of uninhibited peroxidation after the end of the induction period, *R*_ox2_.

## Supporting Information Available

Values of p*K*_a_ for catecholamines; kinetic data for the reaction of L-DOPA, DA, NOR, and ADR with dpph^•^; acid/base equilibrium equations applied for calculation of the degree of ionization of catecholamines; dissociation diagrams of DA, L-DOPA, NOR, and ADR; and kinetic traces for oxidation of liposomes inhibited by PMHC or DA in liposomes (DMPC:DMPG molar ratio of 1:1 or 1:3 or pure DMPG) (PDF)
