# Supplementary material for: Antiradical Activity of Dopamine, L-DOPA, Adrenaline, and Noradrenaline in Water/Methanol and in Liposomal Systems
Source: J Org Chem. 2021 Dec 6;87(3):1791–804. doi: 10.1021/acs.joc.1c02308 (PMC8822484; doi:10.1021/acs.joc.1c02308)
Supplement: Supplementary file 1 — jo1c02308_si_001.pdf [file jo1c02308_si_001.pdf]

Supporting Information

Antiradical Activity of Dopamine, L-DOPA, Adrenaline, and Noradrenaline in Water / Methanol and in Liposomal Systems.

Katarzyna Jodko-Piórecka,[a,]* Bożena Sikora, [a,b] Monika Kluzek, [a,c] Paweł Przybylski,[a] Grzegorz Litwinienko[a,]*

[a] Faculty of Chemistry, University of Warsaw, Pasteura 1, 02-093, Warsaw, Poland
[b] Institute of Physics, Polish Academy of Sciences, Laboratory of Biological Physics, Al. Lotnikow 32/46, 02-668 Warsaw.
[c] Department of Molecular Chemistry and Materials Science, Weizmann Institute of Science, Herzl St 234, Rehovot 76100, Israel

* corresponding authors: kjodko@chem.uw.edu.pl (KJ-P), litwin@chem.uw.edu.pl (GL)

		Page
Table S1.	The values of pK_a for catecholamines.	S2
Table S2.	Kinetic data for the reaction of L-3,4-dihydroxyphenylalanine (L-DOPA) with dpph$^•$.	S3
Table S3.	Kinetic data for the reaction of dopamine (DA) with dpph$^•$.	S4
Table S4.	Kinetic data for the reaction of noradrenaline (NOR) with dpph$^•$.	S5
Table S5.	Kinetic data for the reaction of adrenaline (ADR) with dpph$^•$.	S6
	Acid / base equilibrium equations applied for calculation of the ionization degree of catecholamines.	S7
Figure S1.	Dissociation diagram of DA.	S10
Figure S2.	Dissociation diagram of L-DOPA.	S10
Figure S3.	Dissociation diagram of NOR.	S11
Figure S4.	Dissociation diagram of ADR.	S11
Figure S5.	Kinetic traces for oxidation of DMPC liposomes inhibited by PMHC or DA.	S12
Figure S6.	Kinetic traces for oxidation of DMPC/DMPG 1:1 liposomes inhibited by PMHC or DA.	S12
Figure S7.	Kinetic traces for oxidation of DMPC/DMPG 1:3 liposomes inhibited by PMHC or DA.	S13
Figure S8.	Kinetic traces for oxidation of DMPG liposomes inhibited by PMHC or DA.	S13
Table S6	Kinetic parameters for oxidation of liposomes inhibited by PMHC or DA.	S14
	References.	S14

Table S1. The values of pK_a for DA (determined by *point by point* spectrophotometric analysis in comparison to literature data) and other catecholamines (taken from literature).[a]

Ref	Method	pK_{a0}	pK_{a1}	pK_{a2}	pK_{a3}
**DA**					
this work	*point by point* spectrophotometric analysis		8.37	10.25	12.49
Baba et al.[1]	density functional theory calculations		8.59	11.16	14.08
Sánchez-Rivera et al.[2]	spectrophotometric titration		8.61	9.95	12.04
Antikainen and Witikainen[3]	potentiometric titration		8.85	10.31	
Aydin[4]	potentiometric titration		8.85	10.32	12.62
Kiss et al.[5]	potentiometric titration		8.89	10.41	13.1
Kiss and Gergely[6]	pH-metric titration		8.89	10.41	13.10
Nagy and Takács-Novák[7]	potentiometric titration		8.92	10.50	
Sedeh et al.[8]	potentiometric titration		8.93	10.49	
Rajan et al.[9]	pH-titration		8.96	10.50	
Gh Bagheri[10]	spectrophotometric titration		9.03	-	13.15
Gerard et al.[11]	protometric titration		9.05	10.55	12.81
Sánchez-Rivera et al.[2]	*point by point* analysis		9.05	10.58	12.07
Bretti et al.[12]	potentiometry and spectrofluorimetry		9.06	10.41	
Grgas-Kužnar et al.[13]	pH-titration		9.06	10.60	12.05
Charkoudian and Franz[14]	Combines potentiometric/spectrophotometric		9.59	10.14	13.11
**L-DOPA**					
Martin[15]	potentiometric titration	2.31	8.76	9.84	13.4
Kiss et al.[5]	potentiometric titration		8.80	9.83	13.4
Jameson et al.[16]	^{1}H NMR titration[b]		8.77[b]	9.81[b]	
**NOR**					
Nagy and Takács-Novák[7]	potentiometric titration		8.51	9.63	
Gergely et al.[17]	pH-titration		8.58	9.53	12.9
Aydin[4]	potentiometric titration		8.58	9.53	12.93
**ADR**					
Aydin[4]	potentiometric titration		8.63	9.84	13.13
Gergely et al.[17]	pH-titration		8.64	9.84	13.1
Nagy and Takács-Novák[7]	potentiometric titration		8.66	9.96	
Jameson et al.[16]	^{1}H NMR titration[b]		8.67[b]	9.91[b]	

[a] Values determined by potentiometric titration: for catechol pK_{a1} =9.25 and pK_{a2} =13.0,[18] and for ethanolamine, 2-phenylethylamine, and ethylamine are, respectively, 9.52, 9.89, and 10.68.[16]
[b] Measured in D_2O and recalculated to H_2O.

Table S2. Kinetic data for the reaction of L-DOPA with dpph$^{•}$ determined by stop-flow measurements. Experiments were performed in water / methanol systems (v/v = 1/1) at 23°C, pH 5.5 or pH 7.4 with dpph$^{•}$ at concentrations 28.4 μM and 5.69 μM, respectively. L-DOPA concentrations, [L-DOPA], pseudo-first-order rate constants, k_{exp}, second-order rate constants, k^s, confidence interval of the slope for the 90% confidence level, Δk^s, and coefficients of determination, R^2, are given for each data series. The calculated mean second-order rate constants are given as k^s ± absolute error.

pH 5.5				pH 7.4			
series #1		series #2		series #1		series #2	
[L-DOPA] / mM	k_{exp} 10^3 / s^{-1}	[L-DOPA] / mM	k_{exp} 10^3 / s^{-1}	[L-DOPA] / mM	k_{exp} 10^3 / s^{-1}	[L-DOPA] / mM	k_{exp} 10^3 / s^{-1}
0.517	249	0.517	242	0.0444	3 200	0.0444	2 960
0.402	200	0.402	197	0.0346	2 690	0.0346	2 470
0.313	158	0.313	165	0.0269	2 260	0.0269	2 030
0.235	120	0.235	127	0.0202	1 930	0.0202	1 600
0.176	93.6	0.176	104	0.0151	1 710	0.0151	1 560
0.126	67.7	0.126	84.9	0.0108	1 380	0.0108	1 230
0.0898	53.5	0.0898	66.2	0.00771	1 030	0.00771	943
0.0599	37.8	0.0599	51.8	0.00514	807	0.00514	627
0.0399	27.4	0.0399	38.2			0.00343	429
k^s = 466		k^s = 421		k^s = 58 990		k^s = 58 680	
Δk^s = 7		Δk^s = 16		Δk^s = 5 683		Δk^s = 6 662	
R^2 = 0.9996		R^2 = 0.9972		R^2 = 0.9842		R^2 = 0.9739	
k^s = 440 ± 30 M^{-1} s^{-1}				k^s = 59 000 ± 7 000 M^{-1} s^{-1}			

Table S3. Kinetic data for the reaction of DA with dpph$^\bullet$ determined by stop-flow measurements. Experiments were performed in homogenous water / methanol systems (v/v = 1/1) at 23°C, pH 5.5 or pH 7.4 with dpph$^\bullet$ at concentrations 24.4 µM and 5.27 µM, respectively. DA concentrations, [DA], pseudo-first-order rate constants, k_{exp}, second-order rate constants, k^s, confidence interval of the slope for the 90% confidence level, Δk^s, and coefficients of determination, R^2, are given for each data series. The calculated mean second-order rate constants are given as k^s ± absolute error.

pH 5.5						pH 7.4			
series #1		series #2		series #3		series #1		series #2	
[DA] / mM	k_{exp} 10^3 / s^{-1}	[DA] / mM	k_{exp} 10^3 / s^{-1}	[DA] / mM	k_{exp} 10^3 / s^{-1}	[DA] / mM	k_{exp} 10^3 / s^{-1}	[DA] / mM	k_{exp} 10^3 / s^{-1}
0.591	724	0.459	503	0.349	467	0.0203	3 370	0.0152	2 570
0.459	617	0.345	400	0.262	367	0.0152	2 790	0.0114	2 130
0.345	479	0.185	244	0.187	266	0.0114	2 090	0.00815	1 460
0.258	402	0.132	162	0.134	180	0.00815	1 590	0.00582	1 080
0.185	303	0.0879	118	0.0891	119	0.00582	1 160	0.00388	683
0.132	220	0.0586	76.2	0.0594	77.1	0.00388	797	0.00259	542
0.0879	151					0.00259	553		
0.0586	85.8								
k^s = 1186		k^s = 1062		k^s = 1364		k^s = 162 235		k^s = 167 873	
Δk^s = 113		Δk^s = 80		Δk^s = 73		Δk^s = 10 841		Δk^s = 14 619	
R^2 = 0.9845		R^2 = 0.9941		R^2 = 0.9970		R^2 = 0.9938		R^2 = 0.9920	
k^s = 1 200 ± 200 M^{-1} s^{-1}						k^s = 170 000 ± 10 000 M^{-1} s^{-1}			

Table S4. Kinetic data for the reaction of NOR with dpph$^{\bullet}$ determined by stop-flow measurements. Experiments were performed in homogenous water / methanol systems (v/v = 1/1) at 23°C, pH 5.5 or pH 7.4 with dpph$^{\bullet}$ at concentrations 24.4 μM and 1.46 μM, respectively. NOR concentrations, [NOR], pseudo-first-order rate constants, k_{exp}, second-order rate constants, k^s, confidence interval of the slope for the 90% confidence level, Δk^s, and coefficients of determination, R^2, are given for each data series. The calculated mean second-order rate constants are given as k^s ± absolute error.

pH 5.5				pH 7.4			
series #1		series #2		series #1		series #2	
[NOR] / mM	k_{exp} 10^3 / s^{-1}	[NOR] / mM	k_{exp} 10^3 / s^{-1}	[NOR] / mM	k_{exp} 10^3 / s^{-1}	[NOR] / mM	k_{exp} 10^3 / s^{-1}
0.733	217	0.733	237	0.0376	1 840	0.0292	1 390
0.570	176	0.570	185	0.0292	1 550	0.0227	1 120
0.443	150	0.443	145	0.0227	1 370	0.0171	849
0.333	134	0.333	113	0.0171	1 070	0.0128	679
0.249	102	0.249	97.4	0.0128	868	0.00914	506
0.178	68.7	0.178	70.0	0.00914	636	0.00653	379
0.127	53.1	0.127	52.2	0.00653	488	0.00435	261
0.0848	39.3	0.0848	41.4	0.00435	335	0.00290	183
0.0566	29.1	0.0566	31.8	0.00290	240		
k^s = 278<br>Δk^s = 29<br>R^2 = 0.9776		k^s = 299<br>Δk^s = 9<br>R^2 = 0.9981		k^s = 46 762<br>Δk^s = 4 545<br>R^2 = 0.9807		k^s = 45 550<br>Δk^s = 1 341<br>R^2 = 0.9985	
k^s = 290 ± 30 M^{-1} s^{-1}				k^s = 46 000 ± 5 000 M^{-1} s^{-1}			

Table S5. Kinetic data for the reaction of ADR with $dpph^{\bullet}$ determined by stop-flow measurements. Experiments were performed in homogenous water / methanol systems (v/v = 1/1) at 23°C, pH 5.5 or pH 7.4 with $dpph^{\bullet}$ at concentrations 28.4 μM and 5.48 μM, respectively. ADR concentrations, [ADR], pseudo-first-order rate constants, k_{exp}, second-order rate constants, k^s, confidence interval of the slope for the 90% confidence level, Δk^s, and coefficients of determination, R^2, are given for each data series. The calculated mean second-order rate constants are given as k^s ± absolute error.

pH 5.5				pH 7.4					
series #1		series #2		series #1		series #2		series #3	
[ADR] / mM	k_{exp} 10^3 / s^{-1}	[ADR] / mM	k_{exp} 10^3 / s^{-1}	[ADR] / mM	k_{exp} 10^3 / s^{-1}	[ADR] / mM	k_{exp} 10^3 / s^{-1}	[ADR] / mM	k_{exp} 10^3 / s^{-1}
0.458	342	0.589	512	0.133	3 840	0.0400	1 400	0.0485	1 600
0.357	269	0.458	439	0.104	2 920	0.0311	1 090	0.0377	1 380
0.267	209	0.357	365	0.0806	2 410	0.0242	884	0.0293	1 080
0.201	162	0.201	300	0.0604	1 910	0.0181	744	0.0220	855
0.143	146	0.143	230	0.0453	1 640	0.0136	558	0.0165	714
0.102	123	0.102	210	0.0324	1 160	0.00971	411	0.0118	540
0.0682	83.9			0.0231	919	0.00694	291	0.00841	439
0.0455	65.2			0.0154	648	0.00462	208	0.00561	306
				0.0103	467	0.00308	160	0.00374	198
k^s = 640<br>Δk^s = 47<br>R^2 = 0.9909		k^s = 614<br>Δk^s = 65<br>R^2 = 0.9883		k^s = 26 527<br>Δk^s = 1 243<br>R^2 = 0.9955		k^s = 33 313<br>Δk^s = 1 594<br>R^2 = 0.9953		k^s = 31 300<br>Δk^s = 1 990<br>R^2 = 0.9917	
k^s = 630 ± 60 M^{-1} s^{-1}				k^s = 30 000 ± 3 000 M^{-1} s^{-1}					

Acid / base equilibrium equations applied for calculation of the ionization degree of catecholamines

Dopamine (DA):

Tribasic DA exists in four ionization states, i.e. as a cation ($DA\text{-}H_3^+$), zwitterion ($DA\text{-}H_2$), monoanion ($DA\text{-}H^-$), and dianion (DA^{2-}). The molar fractions of these forms of DA were calculated from equations 1 – 4 with the values of acidity constant pK_{a1} = 8.37, pK_{a2} = 10.25, and pK_{a3} = 12.49, obtained within this work.

$$X(\text{DA-H}_3^+) = \frac{[\text{DA-H}_3^+]}{[\text{DA}]_0} = \frac{[\text{H}^+]^3}{[\text{H}^+]^3 + K_{a1}[\text{H}^+]^2 + K_{a1}K_{a2}[\text{H}^+] + K_{a1}K_{a2}K_{a3}} \quad \text{(S1)}$$

$$X(\text{DA-H}_2) = \frac{[\text{DA-H}_2]}{[\text{DA}]_0} = \frac{K_{a1}[\text{H}^+]^2}{[\text{H}^+]^3 + K_{a1}[\text{H}^+]^2 + K_{a1}K_{a2}[\text{H}^+] + K_{a1}K_{a2}K_{a3}} \quad \text{(S2)}$$

$$X(\text{DA-H}^-) = \frac{[\text{DA-H}^-]}{[\text{DA}]_0} = \frac{K_{a1}K_{a2}[\text{H}^+]}{[\text{H}^+]^3 + K_{a1}[\text{H}^+]^2 + K_{a1}K_{a2}[\text{H}^+] + K_{a1}K_{a2}K_{a3}} \quad \text{(S3)}$$

$$X(\text{DA}^{2-}) = \frac{[\text{DA}^{2-}]}{[\text{DA}]_0} = \frac{K_{a1}K_{a2}K_{a3}}{[\text{H}^+]^3 + K_{a1}[\text{H}^+]^2 + K_{a1}K_{a2}[\text{H}^+] + K_{a1}K_{a2}K_{a3}} \quad \text{(S4)}$$

L-DOPA:

L-DOPA undergoes four-step ionization and five different ionization states can be distinguished depending on pH: cation ($L\text{-}DOPA\text{-}H_4^+$), zwitterion ($L\text{-}DOPA\text{-}H_3$), monoanion ($L\text{-}DOPA\text{-}H_2^-$), dianion ($L\text{-}DOPA\text{-}H^{2-}$), and trianion ($L\text{-}DOPA^{3-}$). Molar fractions of L-DOPA at different ionization states were calculated from equations 5-9 with pK_{a0} = 2.31, pK_{a1} = 8.76, pK_{a2} = 9.84, and pK_{a3} = 13.4 [1].

$$X(\text{L-DOPA-H}_4^+) = \frac{[\text{L - DOPA-H}_4^+]}{[\text{L-DOPA}]_0} = \\ = \frac{[\text{H}^+]^4}{[\text{H}^+]^4 + K_{a0}[\text{H}^+]^3 + K_{a0}K_{a1}[\text{H}^+]^2 + K_{a0}K_{a1}K_{a2}[\text{H}^+] + K_{a0}K_{a1}K_{a2}K_{a3}} \quad \text{(S5)}$$

$$X(\text{L-DOPA-H}_3) = \frac{[\text{L-DOPA-H}_3]}{[\text{L-DOPA}]_0} = \\ = \frac{K_{a0}[\text{H}^+]^3}{[\text{H}^+]^4 + K_{a0}[\text{H}^+]^3 + K_{a0}K_{a1}[\text{H}^+]^2 + K_{a0}K_{a1}K_{a2}[\text{H}^+] + K_{a0}K_{a1}K_{a2}K_{a3}} \tag{S6}$$

$$X(\text{L-DOPA-H}_2^-) = \frac{[\text{L-DOPA-H}_2^-]}{[\text{L-DOPA}]_0} = \\ = \frac{K_{a0}K_{a1}[\text{H}^+]^2}{[\text{H}^+]^4 + K_{a0}[\text{H}^+]^3 + K_{a0}K_{a1}[\text{H}^+]^2 + K_{a0}K_{a1}K_{a2}[\text{H}^+] + K_{a0}K_{a1}K_{a2}K_{a3}} \tag{7}$$

$$X(\text{L-DOPA-H}^{2-}) = \frac{[\text{L-DOPA-H}^{2-}]}{[\text{L-DOPA}]_0} = \\ = \frac{K_{a0}K_{a1}K_{a2}[\text{H}^+]}{[\text{H}^+]^4 + K_{a0}[\text{H}^+]^3 + K_{a0}K_{a1}[\text{H}^+]^2 + K_{a0}K_{a1}K_{a2}[\text{H}^+] + K_{a0}K_{a1}K_{a2}K_{a3}} \tag{S8}$$

$$X(\text{L-DOPA}^{3-}) = \frac{[\text{L-DOPA}^{3-}]}{[\text{L-DOPA}]_0} = \\ = \frac{K_{a0}K_{a1}K_{a2}K_{a3}}{[\text{H}^+]^4 + K_{a0}[\text{H}^+]^3 + K_{a0}K_{a1}[\text{H}^+]^2 + K_{a0}K_{a1}K_{a2}[\text{H}^+] + K_{a0}K_{a1}K_{a2}K_{a3}} \tag{S9}$$

Noradrenaline (NOR):

NOR undergoes four-step ionization and five different ionization states can be distinguished depending on pH: cation (NOR-H_4^+), zwitterion (NOR-H_3), monoanion (NOR-H_2^-), dianion (NOR-H^{2-}), and trianion (NOR^{3-}). The hydroxyl group in the ethylene side chains of NOR is very basic (with $pK_a > 13$)[19] and its deprotonation is neegligible over the evaluate pH range 2-12. Molar fractions of NOR at different ionization states were calculated from equations 10-13 with $pK_{a1} = 8.58$, $pK_{a2} = 9.53$, $pK_{a3} = 12.9$.

$$X(\text{NOR - H}_4^+) = \frac{[\text{NOR - H}_4^+]}{[\text{NOR}]_0} = \frac{[\text{H}^+]^3}{[\text{H}^+]^3 + K_{a1}[\text{H}^+]^2 + K_{a1}K_{a2}[\text{H}^+] + K_{a1}K_{a2}K_{a3}} \tag{S10}$$

$$X(\text{NOR - H}_3) = \frac{[\text{NOR - H}_3]}{[\text{NOR}]_0} = \frac{K_{a1}[\text{H}^+]^2}{[\text{H}^+]^3 + K_{a1}[\text{H}^+]^2 + K_{a1}K_{a2}[\text{H}^+] + K_{a1}K_{a2}K_{a3}} \tag{S11}$$

$$X(\mathrm{NOR\text{-}H_2^-}) = \frac{[\mathrm{NOR\text{-}H_2^-}]}{[\mathrm{NOR}]_0} = \frac{K_{a1}K_{a2}[\mathrm{H^+}]}{[\mathrm{H^+}]^3 + K_{a1}[\mathrm{H^+}]^2 + K_{a1}K_{a2}[\mathrm{H^+}] + K_{a1}K_{a2}K_{a3}} \quad \text{(S12)}$$

$$X(\mathrm{NOR\text{-}H^{2-}}) = \frac{[\mathrm{NOR\text{-}H^{2-}}]}{[\mathrm{NOR}]_0} = \frac{K_{a1}K_{a2}K_{a3}}{[\mathrm{H^+}]^3 + K_{a1}[\mathrm{H^+}]^2 + K_{a1}K_{a2}[\mathrm{H^+}] + K_{a1}K_{a2}K_{a3}} \quad \text{(S13)}$$

Adrenaline (ADR):

ADR undergoes four-step ionization and five different ionization states can be distinguished depending on pH: cation ($ADR\text{-}H_4^+$), zwitterion ($ADR\text{-}H_3$), monoanion ($ADR\text{-}H_2^-$), dianion ($ADR\text{-}H^{2-}$), and trianion (ADR^{3-}). The hydroxyl group in the ethylene side chains of ADR is very basic (with $pK_{a4} > 13$) [2] and its deprotonation is negligible over the evaluate pH range 2-12. Molar fractions of ADR at different ionization states were calculated from equations 14-17 with $pK_{a1} = 8.64$, $pK_{a2} = 9.84$, $pK_{a3} = 13.1$ [3].[17]

$$X(\mathrm{ADR\text{-}H_4^+}) = \frac{[\mathrm{ADR\text{-}H_4^+}]}{[\mathrm{ADR}]_0} = \frac{[\mathrm{H^+}]^3}{[\mathrm{H^+}]^3 + K_{a1}[\mathrm{H^+}]^2 + K_{a1}K_{a2}[\mathrm{H^+}] + K_{a1}K_{a2}K_{a3}} \quad \text{(S14)}$$

$$X(\mathrm{ADR\text{-}H_3}) = \frac{[\mathrm{ADR\text{-}H_3}]}{[\mathrm{ADR}]_0} = \frac{K_{a1}[\mathrm{H^+}]^2}{[\mathrm{H^+}]^3 + K_{a1}[\mathrm{H^+}]^2 + K_{a1}K_{a2}[\mathrm{H^+}] + K_{a1}K_{a2}K_{a3}} \quad \text{(S15)}$$

$$X(\mathrm{ADR\text{-}H_2^-}) = \frac{[\mathrm{ADR\text{-}H_2^-}]}{[\mathrm{ADR}]_0} = \frac{K_{a1}K_{a2}[\mathrm{H^+}]}{[\mathrm{H^+}]^3 + K_{a1}[\mathrm{H^+}]^2 + K_{a1}K_{a2}[\mathrm{H^+}] + K_{a1}K_{a2}K_{a3}} \quad \text{(S16)}$$

$$X(\mathrm{ADR\text{-}H^{2-}}) = \frac{[\mathrm{ADR\text{-}H^{2-}}]}{[\mathrm{ADR}]_0} = \frac{K_{a1}K_{a2}K_{a3}}{[\mathrm{H^+}]^3 + K_{a1}[\mathrm{H^+}]^2 + K_{a1}K_{a2}[\mathrm{H^+}] + K_{a1}K_{a2}K_{a3}} \quad \text{(S17)}$$

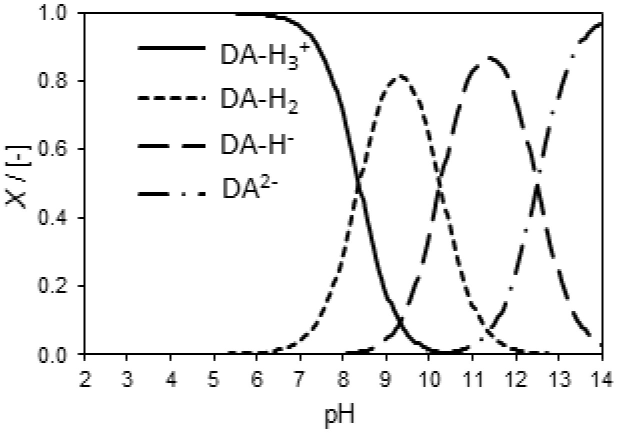

Figure S1. Dissociation diagram of DA over the pH range 2 – 14 obtained from the acid-base equilibrium equations collected in Appendix S1 with the experimental pK_a values obtained within this study. Abbreviations: DA-H_3^+ - cationic DA; DA-H_2 - zwitterionic DA; DA-H^- - DA monoanion; DA^{2-} - DA dianion.

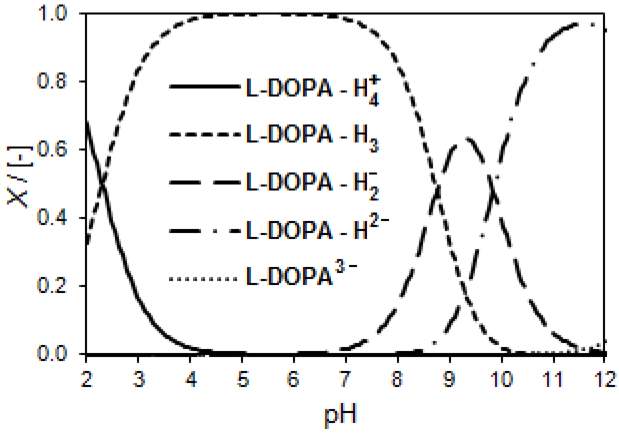

Figure S2. Dissociation diagram of L-DOPA over the pH range 2 – 14 obtained from the acid-base equilibrium equations collected in Appendix S1 with the literature pK_a values [1]. Abbreviations: L-DOPA-H_4^+ - cationic L-DOPA; L-DOPA-H_3 - zwitterionic L-DOPA; L-DOPA-H_2^- - L-DOPA monoanion; L-DOPA-H^{2-} - L-DOPA dianion; L-$DOPA^{3-}$ – L-DOPA trianion.

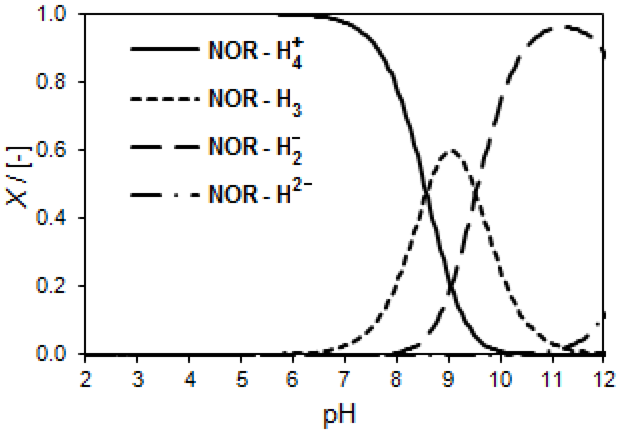

Figure S3. Dissociation diagram of NOR over the pH range 2 – 14 obtained from the acid-base equilibrium equations collected in Appendix S1 with the literature pK_a values [3]. Abbreviations: NOR-H_4^+ - cationic NOR; NOR-H_3 - zwitterionic NOR; NOR-H_2^- - NOR monoanion; NOR-H^{2-} - NOR dianion.

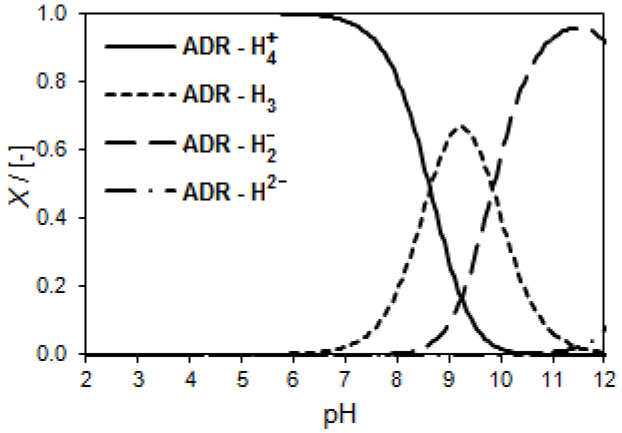

Figure S4. Dissociation diagram of ADR over the pH range 2 – 14 obtained from the acid-base equilibrium equations collected in Appendix S1 with the literature pK_a values [3]. Abbreviations: ADR-H_4^+ - cationic ADR; ADR-H_3 - zwitterionic ADR; ADR-H_2^- - ADR monoanion; ADR-H^{2-} - ADR dianion.

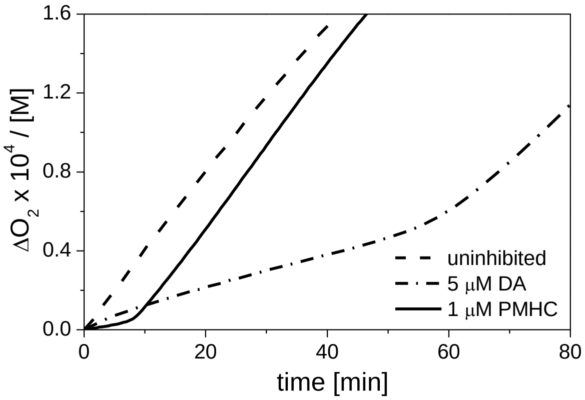

Figure S5. Plots of oxygen uptake, $\Delta[O_2]$, recorded during the peroxidation of MeLin in LUVs composed of DMPC: uninhibited peroxidation proceeding at rate R_{ox1} (dashed line); peroxidation inhibited by 1 μM PMHC (solid line) and 5 μM DA (dash-dotted line). Inhibited processes proceed at the rate R_{inh} during induction period (τ_{ind}) and at the rate R_{ox2} after the end of τ_{ind}. For the values of parameters R_{ox1}, R_{inh}, τ_{ind} and R_{ox2} see Table S6. Experiments were performed at 310 K at pH 7.0 with 10 mM ABAP used to initiate the peroxidation of MeLin.

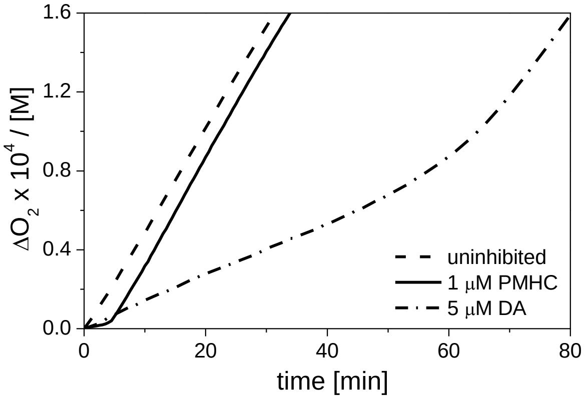

Figure S6. Plots of oxygen uptake, $\Delta[O_2]$, recorded during the peroxidation of MeLin in LUVs composed of DMPC and DMPG at molar ratio 1:1: unihibited peroxidation proceeding at rate R_{ox1} (dashed line); peroxidation inhibited by 1 μM PMHC (solid line) and 5 μM DA (dash-dotted line). Inhibited processes proceed at the rate R_{inh} during induction period (τ_{ind}) and at the rate R_{ox2} after the end of τ_{ind}. For the values of parameters R_{ox1}, R_{inh}, τ_{ind} and R_{ox2} see Table S6. Experiments were performed at 310 K at pH 7.0 with 10 mM ABAP used to initiate the peroxidation of MeLin.

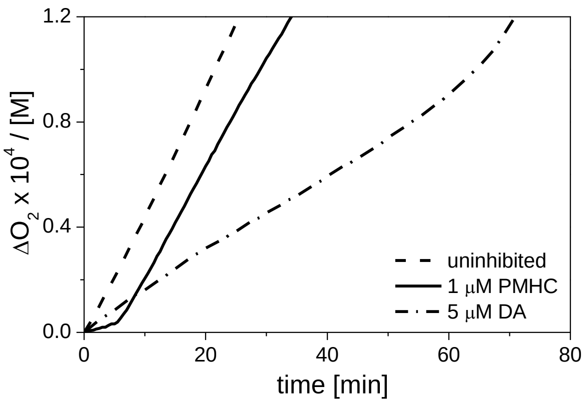

Figure S7. Plots of oxygen uptake, $\Delta[O_2]$, recorded during the peroxidation of MeLin in LUVs composed of DMPC and DMPG at molar ratio 1:3: unihibited peroxidation proceeding at rate R_{ox1} (dashed line); peroxidation inhibited by 1 μM PMHC (solid line) and 5 μM DA (dash-dotted line). Inhibited processes proceed at the rate R_{inh} during induction period (τ_{ind}) and at the rate R_{ox2} after the end of τ_{ind}. For the values of parameters R_{ox1}, R_{inh}, τ_{ind} and R_{ox2} see Table S6. Experiments were performed at 310 K at pH 7.0 with 10 mM ABAP used to initiate the peroxidation of MeLin.

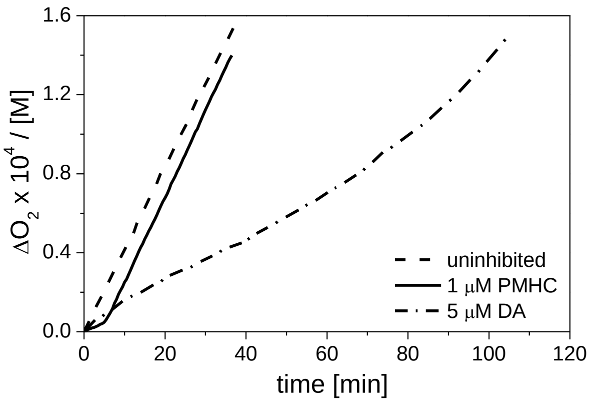

Figure S8. Plots of oxygen uptake, $\Delta[O_2]$, recorded during the peroxidation of MeLin in LUVs composed of DMPG: unihibited peroxidation proceeding at rate R_{ox1} (dashed line); peroxidation inhibited by 1 μM PMHC (solid line) and 5 μM DA (dash-dotted line). Inhibited processes proceed at the rate R_{inh} during induction period (τ_{ind}) and at the rate R_{ox2} after the end of τ_{ind}. For the values of parameters R_{ox1}, R_{inh}, τ_{ind} and R_{ox2} see Table S6. Experiments were performed at 310 K at pH 7.0 with 10 mM ABAP used to initiate the peroxidation of MeLin.

Table S6. Kinetic parameters determined for the peroxidation of 2.74 mM MeLin in DMPC / DMPG LUVs inhibited by 1 μM PMHC or 5 μM DA: the length of induction period, τ_{ind}, the rate of uninhibited peroxidation, R_{ox1}, the rate of inhibited peroxidation, R_{inh} (during the induction period); the rate of peroxidation after the end of induction periods, R_{ox2}. Experiments were performed at 310 K at pH 7.0 with ABAP as an initiator. All numbers represent the average values obtained from series of measurements with calculated standard deviations.

	uninh	1 μM PMHC			5 μM DA		
model	$R_{ox1}\cdot10^8$ / M s^{-1}	τ_{ind} / min	$R_{inh}\cdot10^8$ / M s^{-1}	$R_{ox2}\cdot10^8$ / M s^{-1}	τ_{ind} / min	$R_{inh}\cdot10^8$ / M s^{-1}	$R_{ox2}\cdot10^8$ / M s^{-1}
micelles [a]	42.2 ± 3.3	7.3 ± 0.5	2.1 ± 0.1	26.0 ± 1.2	no antioxidant activity		
LUVs:[b]							
X_{DMPG} 0.00	6.0 ± 0.4	9.6 ± 0.8	1.3 ± 0.2	6.6 ± 0.9	57.0 ± 5.4	1.3 ± 0.1	5.0 ± 0.3
0.25	7.9 ± 0.4	6.0 ± 0.4	1.2 ± 0.1	8.8 ± 0.6	55.7 ± 2.4	2.0 ± 0.3	8.0 ± 0.7
0.50	8.0 ± 0.9	5.9 ± 0.1	1.4 ± 0.2	9.1 ± 0.3	51.2 ± 4.1	2.3 ± 0.2	6.8 ± 0.5
0.75	7.6 ± 1.1	5.9 ± 0.2	1.4 ± 0.2	7.8 ± 1.2	54.9 ± 7.7	2.3 ± 0.2	6.5 ± 0.8
1.00	7.3 ± 0.6	5.3 ± 0.5	1.5 ± 0.2	7.7 ± 0.5	53.3 ± 4.5	2.2 ± 0.1	3.8 ± 0.3

[a] Kinetic parameters obtained for 2.74 mM MeLin peroxidation in Triton X-100 micelles were added for comparison;
[b] LUVs were composed from DMPC and DMPG, with X_{DMPG} – the molar fraction of DMPG in phospholipids: $[n_{DMPG}/(n_{DMPG} + n_{DMPC})]$.

References:

(1) Baba, T.; Matsui, T.; Kamiya, K.; Nakano, M.; Shigeta, Y. A density functional study on the pK_a of small polyprotic molecules *Int. J. Quantum Chem.* **2014**, *114*, 1128-1134.

(2) Sánchez-Rivera, A. E.; Corona-Avendaño, S.; Alarcón-Angeles, G.; Rojas-Hernández, A.; Ramírez-Silva, M. T.; Romero-Romo, M. A. Spectrophotometric study on the stability of dopamine and the determination of its acidity constants *Spectrochim. Acta, Part A* **2003**, *59*, 3193-3203.

(3) Antikainen, P. J.; Witikainen, U. A comparative study on the ionization of catechol amines in aqueous solutions *Acta Chem. Scand.* **1973**, *27*, 2075-2082.

(4) Aydin, R. Study on the interaction of yttrium(III) with adrenaline, noradrenaline, and dopamine *J. Chem. Eng. Data* **2007**, *52*, 2400-2404.

(5) Kiss, T.; Sóvágó, I.; Martin, R. B. Complexes of 3,4-dihydroxyphenyl derivatives. 9. Al^{3+} binding to catecholamines and tiron *J. Am. Chem. Soc.* **1989**, *111*, 3611-3614.

(6) Kiss, T.; Gergely, A. Complexes of 3,4-dhydroxyphnyl derivatives, III. Equilibrium study of parent and some mixed ligand complexes of dopamine, alanine and pyrocatechol with nickel(II), copper(II) and zinc(II) ions *Inorg. Chim. Acta* **1979**, *36*, 31-36.

(7) Nagy, P. I.; Takács-Novák, K. Tautomeric and conformational equilibria of biologically important (hydroxyphenyl)alkylamines in the gas phase and in aqueous solution *PCCP* **2004**, *6*, 2838-2848.

(8) Sedeh, I. F.; Sjöberg, S.; Öhman, L. O. Equilibrium and structural studies of silicon(IV) and aluminum(III) in aqueous solution. 31. Aqueous complexation between silicic acid and the catecholamines dopamine and L-DOPA *J. Inorg. Biochem.* **1993**, *50*, 119-132.

(9) Rajan, K. S.; Davis, J. M.; Colburn, R. W. Metal chelates in the storage and transport of neurotransmitters: interactions of metal ions with biogenic amines *J. Neurochem.* **1971**, *18*, 345-364.

(10) Gh Bagheri, A. Thermodynamic Complexation of Dopamine with Molybdenum (VI) in Media with Different Dielectric Constants *J. Chem. Eng. Data* **2009**, *54*, 2981-2985.

(11) Gerard, C.; Chehhal, H.; Hugel, R. P. Complexes of iron(III) with ligands of biological interest: dopamine and 8-hydroxyquinoline-5-sulphonic acid *Polyhedron* **1994**, *13*, 541-597.

(12) Bretti, C.; Crea, F.; De Stefano, C.; Foti, C.; Materazzi, S.; Vianelli, G. Thermodynamic properties of dopamine in aqueous solution. Acid-base properties, distribution, and activity coefficients in NaCl aqueous solutions at different ionic strengths and temperatures *J. Chem. Eng. Data* **2013**, *58*, 2835-2847.

(13) Grgas-Kužnar, B.; Simeon, V.; Weber, O. A. Complexes of adrenaline and related compounds with Ni^{2+}, Cu^{2+}, Zn^{2+}, Cd^{2+} and Pb^{2+} *J. Inorg. Nucl. Chem.* **1974**, *36*, 2151-2154.

(14) Charkoudian, L. K.; Franz, K. J. Fe(III)-Coordination Properties of Neuromelanin Components: 5,6-Dihydroxyindole and 5,6-Dihydroxyindole-2-carboxylic Acid *Inorg. Chem.* **2006**, *45*, 3657-3664.

(15) Martin, B. R. Zwitterion formation upon deprotonation in l-3,4-dihydroxyphenylalanine and other phenolic amines *J. Phys. Chem.* **1971**, *75*, 2657-2661.

(16) Jameson, R. F.; Hunter, G.; Kiss, T. A 1H nuclear magnetic resonance study of the deprotonation of L-dopa and adrenaline *J. Chem. Soc., Perkin Trans. 2* **1980**, 1105-1110.

(17) Gergely, A.; Kiss, T.; Deák, G.; Sóvágó, I. Complexes of 3,4-dihydroxyphenyl derivatives IV. Equilibrium studies on some transition metal complexes formed with adrenaline and noradrenaline *Inorg. Chim. Acta* **1981**, *56*, 35-40.

(18) Balla, J.; Kiss, T.; Jameson, R. F. Copper (II)-catalyzed oxidation of catechol by molecular oxygen in aqueous solution *Inorg. Chem.* **1992**, *31*, 58-62.

(19) Jameson, R. F.; Neillie, W. F. S. 439. Complexes formed by adrenaline and related compounds with transition-metal ions. Part I. Acid dissociation constants of the ligands *J. Chem. Soc.* **1965**, 2391-2395.
